# On the Scope of Lagrangian Vortex Methods for Two-Dimensional Flow Simulations and the POD Technique Application for Data Storing and Analyzing

**DOI:** 10.3390/e23010118

**Published:** 2021-01-18

**Authors:** Kseniia Kuzmina, Ilia Marchevsky, Irina Soldatova, Yulia Izmailova

**Affiliations:** 1 Ivannikov Institute for System Programming of the Russian Academy of Sciences, 109004 Moscow, Russia; kuz-ksen-serg@yandex.ru (K.K.); i-soldatova@bk.ru (I.S.); 2 Department of Applied Mathematics, Bauman Moscow State Technical University, 105005 Moscow, Russia; yulia.izmailova@mail.ru

**Keywords:** vortex particle method, viscous vortex domain method, proper orthogonal decomposition, open source code, Blasius boundary layer, impulsively started cylinder, channel with backward facing step

## Abstract

The possibilities of applying the pure Lagrangian vortex methods of computational fluid dynamics to viscous incompressible flow simulations are considered in relation to various problem formulations. The modification of vortex methods—the Viscous Vortex Domain method—is used which is implemented in the VM2D code developed by the authors. Problems of flow simulation around airfoils with different shapes at various Reynolds numbers are considered: the Blasius problem, the flow around circular cylinders at different Reynolds numbers, the flow around a wing airfoil at the Reynolds numbers $10^{4}$ and $10^{5}$, the flow around two closely spaced circular cylinders and the flow around rectangular airfoils with a different chord to the thickness ratio. In addition, the problem of the internal flow modeling in the channel with a backward-facing step is considered. To store the results of the calculations, the POD technique is used, which, in addition, allows one to investigate the structure of the flow and obtain some additional information about the properties of flow regimes.

## 1. Introduction

Flow simulation problems and, more generally, computational fluid dynamics (CFD) problems, are among the most difficult problems of computational mathematics, mainly due to the nonlinearity of the governing equations and the need to take into account a large number of factors which have both a physical nature (heat transfer, convection, chemical reactions, cavitation, etc.), and are related to the requirements of the necessary properties of the numerical scheme, such as accuracy, conservatism, stability, monotony, etc.

There is, however, a rather wide range of problems, for example, fluid–structure interaction (FSI) problems, where it is necessary to estimate hydro- or aerodynamic loads acting on the structure. At the same time, the flow simulation in most cases is a much more complicated subtask in comparison to a structural solver. So, it is important to develop numerical methods that permit aerohydrodynamic loads estimation with acceptable accuracy and as low as possible computational cost. The flow itself in such problems is usually out of direct interest, but it is necessary to simulate it in order to compute correctly pressure distribution and viscous friction over the body surface, which are the source of integral forces and momenta. In other applications, i.e., in industrial aerodynamics it is also important to describe some “rough” (large-scale) properties of the flow: to predict zones of increased flow velocity, stagnant zones, large vortex structures. Similar problems arise also in applications connected with particle simulation in the flows: usually, it is possible to “uncouple” the problem and to solve the aerohydrodynamic problem separately, while particle motion is modelled in preliminarily obtained velocity and pressure fields (sometimes computed even in a steady-state statement).

In addition to the fact that the problems of numerical flow simulation are traditionally time-consuming, in many cases, the problem of storing and analyzing the simulation results is actual, especially when an unsteady flow is simulated and it is required to store snapshots at many time steps. This problem sometimes wrongly seems to be not essential or at least much more simple in comparison to flow numerical simulation itself, however, in practice, it has nearly the same importance. Computational meshes in real engineering problems that are currently relevant may contain millions of cells, and the number of time steps may have the same order. Even simple storing of computational results, i.e., velocity and pressure, as well as other physical fields, in nodes/mesh cells with a certain time step requires a huge amount of disk space, and their further processing also requires significant amounts of RAM. In many cases, the effects of interest are large-scale, so the spatial resolution of the obtained results (which is determined by the computational mesh and time step) is superfluous for practical purposes when processing and interpreting them, but it is necessary to obtain a qualitatively and quantitatively correct solution.

In the present paper, we consider the so-called vortex particle methods (VPM), confining ourselves to two-dimensional problem statements. Despite the rather narrow range of applicability, which will be outlined below, such methods can be extremely efficient in engineering applications, when it is necessary to estimate unsteady loads acting on the structure and to predict the most significant properties of the flow, such as large vortex structures, etc.

The aim of this paper is to describe some modern modifications of two-dimensional VPMs, the use of which made it possible to improve the accuracy of flows simulations significantly, and to show the efficiency of the proper orthogonal decomposition (POD) technique for the results of flow simulations storing and further reconstruction, as well as for different flow regimes distinguishing.

## 2. On Two-Dimensional Vortex Methods and Their Range of Applicability

In two particular cases—steady-state regimes simulation and, in opposite, essentially unsteady oscillations (vibrations)—the simplified approaches for hydrodynamic loads estimating can be implemented. For steady-state flows, when the body is immovable or moves rather slow, stationary dimensionless coefficients of drag and lift force and momenta can be used, which values are known from experiment or numerical simulation, being performed preliminary. These coefficients depend on the body shape, its orientation (angle of attack, AoA), and usually the Reynolds number. The other approach that follows from the added mass theory, can be applied, for example, for vibrating bodies, when the aerohydrodynamic loads are determined mainly by the accelerated motion of the body in media while the pressure distribution and viscous friction a fortiori provide a negligible contribution.

The last approach is closely connected to another case when the flow is considered to be potential. Accepting the additional assumption about the incompressibility of the media, the problem of flow simulation comes to solving the Laplacian equation with respect to scalar potential function, which gradient is flow velocity, while the pressure and forces can be reconstructed according to the Bernoulli or Cauchy–Lagrange integrals. This simplification, in turn, does now allow to take into account the viscosity influence, and in particular to provide the no-slip boundary condition satisfaction on the body surface that leads to vorticity generation, flow separation, etc. So, the potential flow simplification can be used only in very rough models.

However, we should note that in the framework of the above-mentioned approach it is possible to avoid the solving of the Laplacian equation in the whole flow domain and to replace it by solving the boundary integral equation (BIE) with respect to simple layer intensity, double layer intensity, or vortex sheet intensity, located on the body surface. This approach is especially suitable in the case of outer flows simulation when the flow domain is unbounded; it allows for exact satisfaction of the continuity (incompressibility) equation and the perturbation decay boundary condition on infinity and does not require some “artificial” boundary condition of non-reflective type.

These ideas were assumed as a basis of the first generation of the so-called vortex particle methods (VPM), which could be applied for two-dimensional flow simulation around thin plates and wing airfoils when separation point is specified a priori at the edges or corner points. Some modifications of such approach, called discrete vortices method (DVM), are still used in corresponding applications even nowadays, mainly due to the extreme simplicity of the numerical algorithm. A detailed review of the first generation of the vortex methods can be found in [1,2].

At the same time, modern modifications of VPM have been developed that allow for viscous incompressible flows simulation that are described by the Navier–Stokes equations. At least three approaches are known to viscosity effect taking into account, namely random walk method [3], particle strength exchange (PSE) method [4], and diffusive velocity method. The last one initially suggested in [5] and then improved significantly in [6,7,8] seems to be the most accurate, however, its implementation is non-trivial. The VPM modification based on this approach is called the viscous vortex domains method (VVD). The computational cost of its algorithms, of course, is much higher in comparison to DVM, but they remain competitive in comparison to traditional mesh-based numerical methods, especially in coupled FSI problems, due to the fully Lagrangian nature of the VPM: they are meshless, and there is no need to reconstruct somehow a mesh following to the body motion [9].

In the framework of the random walk method, an efficient implementation of vortex methods is developed in [10] that is applied efficiently to industrial aerodynamics [11].

At last, it is clear that the consideration of two-dimensional problems is extremely attractive from the computational point of view, so it is important to determine the conditions at which the two-dimensional problem statement is correct. In the present paper, we consider some two-dimensional model problems and the results of simulation by using VPM. The results will be compared to known experimental data and numerical results, obtained by other authors. Real flows (in experiments) are considered around the cylindrical bodies which cross-section coincides with the airfoils in two-dimensional problems. It is well-known that there is some critical value of the Reynolds number when the flow becomes turbulent that means that it is essentially three-dimensional, at least, on microscales, however, it affects significantly the parameters of the macroscale flow (which can remain two-dimensional). In traditional mesh-based methods, such effects are normally taken into account in the framework of Reynolds-averaging technique (RANS) of Large Eddy Simulation (LES) by considering averaged Navier–Stokes equations together with some closure equations, called turbulence models.

For VPM there are no known “turbulence models” nowadays, and it is important to outline the boundaries when two-dimensional simulation remains correct and it is in acceptable agreement with the results of full-scale experiments. However, it is interesting to note that in [12,13] the hybrid approach is suggested, based on the control-volume method and an original simplified modification of vortex methods at subgrid scale in order to reproduce turbulent pulsations in the flow field.

At the end of the VPM general description, we note that among the particle-based methods, smoothed-particle hydrodynamics (SPH) is the most popular and well-known family of methods that can be applied for a wide range of simulations in CFD. The review of the current state of the SPH can be found at [14]. However, since in SPH the particles are material and they represent some volumes of the simulated medium and are characterized by masses (or densities), the particles must fill the entire flow domain, while vorticity, and hence, the vortex particles modeling it in VPM, as a rule, occupy only a relatively small area (vortex wake) near the surface and behind the body. In addition, vortex particles have the property called “action at a distance”: all the vortex particles influence all others. Although such influence decreases with increasing distance, it can not be neglected even for far enough placed particles. At the same time, in SPH the effect of particles is taken into account only at a small distance, which has an order of doubled smoothing length. So, despite the fact that both SPH and VPM belong to Lagrangian particle-based methods, there are fundamental differences between them.

## 3. Brief Description of the Viscous Vortex Domains Method for Two-Dimensional Incompressible Flow Simulation

Two-dimensional viscous incompressible flows are described by the Navier–Stokes equations

(1) $$\nabla \cdot \vec{V}=0,\;\frac{\partial \vec{V}}{\partial t}+\left(\vec{V}\cdot \nabla \right)\vec{V}=-\frac{\nabla p}{\rho }+\nu \Delta \vec{V},$$

where $\vec{V}$ is flow velocity, *p* is pressure, ρ and ν are density and kinematic viscosity of the media, respectively. In the framework of Lagrangian VPM, vorticity $\vec{\Omega }=\nabla \times \vec{V}$ is a primary computational variable instead of the so-called “primitive” variables $\vec{V}$ and *p* being considered in Eulerian mesh methods.

Flow velocity field for the media in the most general case can be reconstructed according to the generalized Biot–Savart law [15,16,17,18]:

(2) $$\begin{array}{c} \vec{V}\left(\vec{r}\right)={\vec{V}}_{\infty }+\int_{S}\frac{\vec{\Omega }\left(\vec{\xi }\right)\times \left(\vec{r}-\vec{\xi }\right)}{2\pi |\vec{r}-\vec{\xi }{|}^{2}}dS_{\xi }+\int_{S}\frac{D\left(\vec{\xi }\right)\left(\vec{r}-\vec{\xi }\right)}{2\pi |\vec{r}-\vec{\xi }{|}^{2}}dS_{\xi }\\ \;+\oint_{K}\frac{\left(\vec{n}\left(\vec{\xi }\right)\cdot {\vec{V}}_{K}\left(\vec{\xi }\right)\right)\left(\vec{r}-\vec{\xi }\right)}{2\pi |\vec{r}-\vec{\xi }{|}^{2}}dl_{\xi }+\oint_{K}\frac{\left(\vec{n}\left(\vec{\xi }\right)\times {\vec{V}}_{K}\left(\vec{\xi }\right)\right)\times \left(\vec{r}-\vec{\xi }\right)}{2\pi |\vec{r}-\vec{\xi }{|}^{2}}dl_{\xi }. \end{array}$$

Here, ${\vec{V}}_{\infty }$ is incident flow velocity; $D(\vec{\xi })$ is velocity divergence, which is equal to zero in case of incompressible media being considered in VPM; *S* is flow domain; *K* is its boundary (the union of the airfoil boundaries); ${\vec{V}}_{K}\left(\vec{\xi }\right)$ is the velocity of the corresponding point of the boundary; $\vec{n}\left(\vec{\xi }\right)$ is outer normal unit vector.

Pressure distribution in the flow domain can be reconstructed by using the analogue of the Bernoulli and the Cauchy–Lagrange integrals [19,20]. If it is necessary to estimate integral force and momenta acting on the airfoils in the flow, it is possible to avoid the pressure calculation and use rather simple formulae for total force and momenta that can be written down in different forms depending on the problem being solved [21,22].

For the velocity field described by (2), the divergence-free condition is satisfied automatically, while the Navier–Stokes equation, after applying the curl operator, in the two-dimensional case can be written down as the following:

(3) $$\frac{\partial \vec{\Omega }}{\partial t}+\nabla \times \left(\vec{V}+\vec{W}\right)=0,$$

where $\vec{W}$ is the so-called diffusive velocity, which expresses the viscosity effect. Because of considering two-dimensional flows, vorticity field $\vec{\Omega }$ is fully described by scalar field Ω, such as $\vec{\Omega }=\Omega \vec{k}$, where $\vec{k}$ is a unit vector orthogonal to the flow plane. Taking it into account, rather simple expression for diffusive velocity can be written down:

$$\vec{W}=-\nu \frac{\nabla \Omega }{\Omega }.$$

Equation (3) means that the vorticity evolution in the flow domain can be considered as its transfer along the streamlines of summary velocity field $\left(\vec{V}+\vec{W}\right)$. In numerical simulation vorticity field is modelled with a large number of Lagrangian vortex particles; each particle is described by its position ${\vec{r}}_{k}$ and strength $\Gamma_{k}$, which is a circulation of velocity field along the closed contour encircling the particle. Particles have velocities $\left({\vec{V}}_{k}+{\vec{W}}_{k}\right)$, and numerical algorithm for diffusive velocities computation is suggested in [8], where it is taken as a basis for the viscous vortex domains (VVD) method. Note that the diffusive velocities being computed straightforwardly according to [8], sometimes suffer from overshoots; so in the simulations presented in this paper the maximal magnitude of the diffusive velocities was bounded by the value of $1.5V_{\infty }$.

The governing Equation (1) are supplemented with the boundary conditions of perturbation decay at infinity (if the flow domain is unbounded, otherwise the pressure value should be specified at some point)

$$\vec{V}\left(\vec{r}\right)\to {\vec{V}}_{\infty },\;p\left(\vec{r}\right)\to p_{\infty }\;at\;\left|\vec{r}\right|\to \infty ,$$

and the no-slip boundary conditions on the walls (airfoil boundaries):

$$\vec{V}\left(\vec{r}\right)={\vec{V}}_{K}\left(\vec{r}\right)\;at\;\vec{r}\in K.$$

The perturbation decay conditions at infinity are satisfied automatically in vortex methods, which makes it possible to avoid artificial bounding of the flow domain. However, considering the infinite flow domain does not affect the computational complexity of the simulation: since the primary computational variable is vorticity, and it is normally localized in some compact region nearby the airfoil and behind it, the computational resources are localized in the vortex wake area.

## 4. High-Precision Numerical Schemes for Vorticity Generation Simulating

The no-slip boundary condition satisfaction is provided by vorticity generation on the walls (airfoil boundaries). Note that it is impossible to derive physically correct boundary condition with respect to vorticity, so it is formulated for the “primitive” variable, i.e., velocity field [18,23,24]; it therefore has integral form in terms of vorticity. The straightforward way to its formulation is considering the boundary integral equation (BIE) with respect to the vorticity flux on the airfoil surface [25,26], but such an approach is not very suitable from a practical point of view. So, it seems to be useful to accept the following simplification: considering a small time period Δt, assume that the vorticity being generated during it fills a thin layer in proximity to the airfoil boundary. Then, in turn, we neglect its width and consider it as a thin vortex sheet of unknown intensity $\gamma (\vec{r})$, $\vec{r}\in K$. This model can be also treated as implicit numerical scheme implementation according to which the boundary condition satisfaction is provided at the end of the time step (instead of its beginning in case of the BIE solving with respect to the vortex flux that corresponds to the explicit numerical scheme) that leads to numerically stable algorithm even for large time steps Δt.

The BIE that follows directly from the no-slip condition is a vector equation since it expresses the equality of boundary value of velocity field to the wall velocity. It can be replaced with equivalent scalar BIE: expressing the equality between normal and tangent velocity components. The first way, called “*N*-model”, is well-known and widely used [27,28,29]. The second one, “*T*-model”, is described in general words in [18,23] and mentioned in [28,30]. However, this approach is not used in existing implementations of vortex methods, possibly due to the fact that the straightforward transfer of the discretization procedure from the *N*-model onto *T*-models does not lead to accuracy increasing at all. At the same time, improved discretization that can be applied only to the *T*-models makes it possible to increase the accuracy significantly.

Note that while the *N*-model is expressed by a singular BIE of the 1-st kind with a non-integrable kernel of the Hilbert-type, where integral should be understood as Cauchy principal value [28,31,32], the equation in the *T*-model is the Fredholm-type BIE of the 2-nd kind with bounded or at least absolutely integrable kernel.

The unknown variable is a vortex sheet intensity $\gamma (\vec{r})$ being generated at the airfoil boundary during small time step Δt:

(4) $$\oint_{K}Q_{\tau }\left(\vec{r},\vec{\xi }\right)\gamma \left(\vec{\xi }\right)dl_{\xi }-\frac{1}{2}\gamma \left(\vec{r}\right)=f_{\tau }\left(\vec{r}\right),\;Q_{\tau }\left(\vec{r},\vec{\xi }\right)=\frac{\vec{n}\left(\vec{r}\right)\cdot \left(\vec{r}-\vec{\xi }\right)}{2\pi |\vec{r}-\vec{\xi }{|}^{2}},$$

which right-hand side has the following form:

(5) $$\begin{array}{c} f_{\tau }\left(\vec{r}\right)=\vec{\tau }\left(\vec{r}\right)\cdot (\frac{1}{2}{\vec{V}}_{K}\left(\vec{r}\right)-{\vec{V}}_{\infty }-\int_{S}\frac{\vec{k}\times \left(\vec{r}-\vec{\xi }\right)}{2\pi |\vec{r}-\vec{\xi }{|}^{2}}\Omega \left(\vec{\xi }\right)\;dS_{\xi }\\ \;-\oint_{K}\frac{\left(\vec{n}\left(\vec{\xi }\right)\times {\vec{V}}_{K}\left(\vec{\xi }\right)\right)\times \left(\vec{r}-\vec{\xi }\right)}{2\pi |\vec{r}-\vec{\xi }{|}^{2}}\;dl_{\xi }-\oint_{K}\frac{\left(\vec{n}\left(\vec{\xi }\right)\cdot {\vec{V}}_{K}\left(\vec{\xi }\right)\right)\left(\vec{r}-\vec{\xi }\right)}{2\pi |\vec{r}-\vec{\xi }{|}^{2}}\;dl_{\xi }), \end{array}$$

where $\Omega (\vec{\xi })$ is vorticity distribution in the flow domain.

We will not describe in details the properties of the integral operator in the Equation (4) and the right-hand side (5), which depend on the type of problem being solved. The only thing that should be noted that the Equation (4) has an infinite set of solutions if an unbounded flow domain is considered, so in order to select the unique physically correct solution, an additional integral condition for the total vorticity should be added:

(6) $$\oint_{K}\gamma \left(\vec{\xi }\right)dl_{\xi }=\Gamma_{*},$$

and if *K* is a union of airfoils $K_{i}$, the conditions similar to (6) should be written down for each airfoil.

We should note that in [18,23] it is wrongly asserted that (4) has a unique solution; precisely speaking, it is true, but only for internal flows in bounded domains.

For the coupled FSI problems the right-hand side (5) depends on unknown airfoil boundary velocity ${\vec{V}}_{K}\left(\vec{r}\right)$, so some type of splitting (or coupling) scheme should be implemented—an intuitive weak coupling scheme (step-by-step flow simulation step for the airfoil moving according to known law, and structure motion step for the airfoil moving under known forces), fully monolithic scheme [33] or iterative scheme [25]. The last approach requires added mass tensor calculation for the airfoil and can be characterized as a strongly-coupled scheme; it seems to be the most robust, and it is preferable for practical purposes.

The family of numerical schemes for approximate solution of the BIE (4) with additional equations required for the selection of the unique solution, that have been developed on the basis of the Galerkin method, is described in [34,35,36]. Such schemes are called “*T*-schemes” and they are in general case much more accurate in comparison to the “*N*-schemes” traditionally used for solving equivalent singular BIE instead of (4). Note that the developed approach can be generalized to the three-dimensional case [37]. An efficient iterative approach to solving of the resulting linear systems is suggested in [38].

As the result, vorticity distribution in the flow domain is modelled by large number of vortex particles being considered as point (circular) vortices of small radius ε, having circulations $\Gamma_{i}$, which are placed at points ${\vec{r}}_{i}$, and move according to system of ordinary differential equations

(7) $$\frac{d{\vec{r}}_{i}}{dt}=\vec{V}\left({\vec{r}}_{i}\right)+\vec{W}\left({\vec{r}}_{i}\right),\;i=1,\;\ldots ,\;N.$$

Circulations $\Gamma_{i}$ remain constant; they can only change at vortex particles merging.

Vorticity generated and concentrated in the vortex sheet on the airfoil boundary at the next time step is shrunk into a set of vortex particles, placed in proximity to the boundary. As a rule, the maximal value of vortex particle circulation is bounded by some value $\Gamma_{\max}$. Particles in the flow placed on rather small distance, are merged into one particle, however, the merging is performed only when the circulation of the resulting particle is smaller than $\Gamma_{\max}$. This procedure makes it possible to reduce the number of vortex particles significantly, which reduces, in turn, the total computational complexity of the whole algorithm. Furthermore, at flow simulation after the airfoils, both well-streamlined and bluff, far vortex wake can be thrown off, because it practically does not influence the forces acting on the airfoil and the flow near to it.

The last point that should be mentioned, is connected with the “no-penetration” condition control: vortex particles that move in the neighborhood of the airfoil boundary according to (7), due to numerical errors, caused both by finite time step and errors in velocities computation, sometimes cross the airfoil boundary. Such particles should be found and excluded from the simulation, of course, with adding corresponding contributions to other equations to provide conservation properties, etc.

## 5. Added Masses Tensor Components Estimation

In order to verify the developed algorithms for the boundary integral equation solution, we consider the model problem with a known exact solution. However, it seems to be useful not to estimate the accuracy in a purely mathematical sense by calculating some norm of the difference between exact and approximate solution, but to calculate some integral characteristic of the solution. Namely, we calculate all the components of the added mass tensor of the airfoil [39,40,41]. These values are required in particular in vortex methods implementation for numerical simulation in coupled FSI problems [25], and also they can be used directly in approximate engineering techniques for estimation of the forces acting on the body that moves with non-zero acceleration [42].

**Figure 1:**
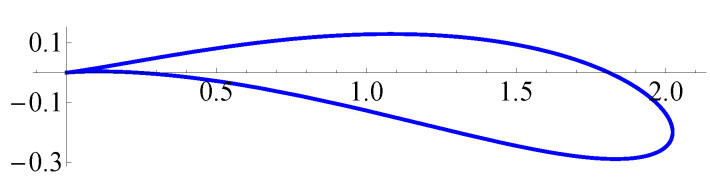
Zhukovsky airfoil shape on ζ complex plane for a=1, η=1.15, α=π/30.

The exact values for added masses are known for some simple-shaped airfoils. One of the most powerful approaches for its exact computation is the usage of the conformal mappings technique. For example, if we consider complex plane *z* and map a circle of radius ηa which center is placed at point $z_{c}=a\left(\eta e^{i\alpha }-1\right)$ onto complex plane ζ according to the rule

$$\zeta \left(z\right)=e^{-i\alpha }\left(z+\frac{1}{2}\left(z+\frac{a^{2}}{z}\right)\right),$$

we obtain the Zhukovsky airfoil shape [41,43] which specific feature is the cusp at the trailing edge at point ζ=0 (Figure 1).

The exact expressions can be written down for all the components of the added mass tensor which is symmetrical (here σ=η/(2ηcosα−1), ρ is density of the media), [41]:

$$\begin{array}{c} \lambda_{xx}^{*}=\frac{\pi \rho a^{2}}{4}\left(\sigma^{2}+\eta^{2}-2\cos2\alpha \right),\;\lambda_{yy}^{*}=\frac{\pi \rho a^{2}}{4}\left(\sigma^{2}+\eta^{2}+2\cos2\alpha \right),\;\lambda_{xy}^{*}=\frac{\pi \rho a^{2}}{2}\sin2\alpha ,\\ \lambda_{x\omega }^{*}=\frac{\pi \rho a^{3}}{8}\sin\alpha \left(\sigma^{2}+\eta^{2}+4\left(\sigma +\eta \right)\cos\alpha \right),\\ \lambda_{y\omega }^{*}=\frac{\pi \rho a^{3}}{8}\left(\sigma^{3}+\eta^{3}+\left(\sigma^{2}+\eta^{2}\right)\cos\alpha +2\left(\sigma +\eta \right)\cos2\alpha \right),\\ \lambda_{\omega \omega }^{*}=\frac{\pi \rho a^{4}}{8}\sigma^{2}\eta^{2}\left(8\sigma^{2}\eta^{2}\cos^{4}\alpha -2\sigma \eta \sin^{2}2\alpha +\cos4\alpha \right). \end{array}$$

In [25], it is shown that the components of the added mass tensor could be easily calculated if solution $\gamma (\vec{\xi })$ of the BIEs (4)–(6) is known. To do it, one should consider the model problems: impulsive start of the airfoil in horizontal and vertical directions in still media with unit velocity $|{\vec{V}}_{K}|=1$, and its impulsive start in rotational motion with unit angular velocity ω=1.

In addition to the vortex sheet intensity $\gamma (\vec{\xi })$ it is suitable to introduce the attached vortex sheet intensity which is equal to tangent velocity component of the airfoil surface velocity:

$$\tilde{\gamma }\left(\vec{r}\right)={\vec{V}}_{K}\left(\vec{r}\right)\cdot \vec{\tau }\left(\vec{r}\right),\;\vec{r}\in K,$$

where $\vec{\tau }\left(\vec{r}\right)$ is tangent unit vector, which direction is chosen such as $\vec{n}\left(\vec{r}\right)\times \vec{\tau }\left(\vec{r}\right)=\vec{k}$; $\vec{n}\left(\vec{r}\right)$ is unit outer normal vector (directed to the flow domain); $\vec{k}$ is unit vector orthogonal to the flow plane. The value of total circulation in additional condition (6) should be chosen as

$$\Gamma_{*}=-\oint_{K}\tilde{\gamma }\left(\vec{\xi }\right)dl_{\xi },$$

it is non-zero only in rotational motion.

So, the components of the added mass tensor for two-dimensional flow are calculated as follows [25]:

$$\begin{array}{c} \lambda_{dx}=\oint_{K}\rho y\left(\gamma_{d}\left(\vec{r}\right)+{\tilde{\gamma }}_{d}\left(\vec{r}\right)\right)dl,\;\lambda_{dy}=-\oint_{K}\rho x\left(\gamma_{d}\left(\vec{r}\right)+{\tilde{\gamma }}_{d}\left(\vec{r}\right)\right)dl,\\ \lambda_{d\omega }=-\frac{1}{2}\oint_{K}\rho \left(x^{2}+y^{2}\right)\left(\gamma_{d}\left(\vec{r}\right)+{\tilde{\gamma }}_{d}\left(\vec{r}\right)\right)dl, \end{array}$$

where *x* and *y* are the abscise and the ordinate of the point $\vec{r}$ at the airfoil boundary; symbol *d* has values *x*, *y* and ω; $\gamma_{d}\left(\vec{\xi }\right)$ and ${\tilde{\gamma }}_{d}\left(\vec{\xi }\right)$ denote vortex sheets intensities that correspond to the airfoil motion in the *d*-th direction.

In the numerical experiment, all nine components of the added mass tensor were calculated, and the relative errors in comparison to exact values were estimated. Three numerical schemes have been considered:

- Classical well-known numerical scheme of the Discrete Vortex Method [27,28] that belongs to the above discussed *N*-schemes and corresponds to numerical solving of the singular BIE (*N*-model);
- Developed $T^{0}$-scheme, according to which the numerical solution is assumed to be piecewise-constant [35,36], and the 2nd kind Fredholm-type integral Equation (4) is solved;
- Developed $T^{1}$-scheme, according to which the numerical solution is assumed to be piecewise-linear [34,36].

The surface line of the airfoil was discretized and replaced with a polygon with rectilinear legs, usually called “panels” which have nearly the same length. In Table 1, maximal relative errors are shown for all the components of the added mass tensor for a different number of the panels. The error values are computed as follows:

$$\delta =\max_{i,j}\frac{\left|\lambda_{i,j}-\lambda_{i,j}^{*}\right|}{\lambda_{i,j}^{*}},\;i,\;j=x,\;y,\;\omega .$$

**Figure 2:**
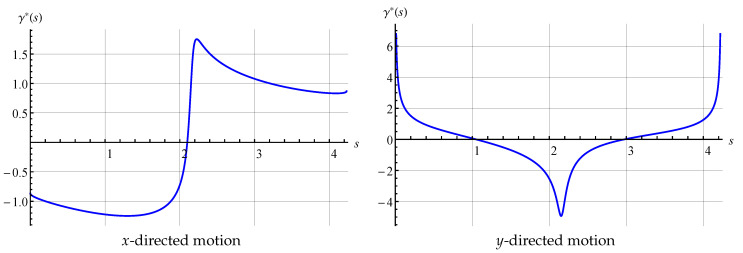
Exact solution for the vortex sheet intensity $\gamma^{*}\left(s\right)$ for the Zhukovsky wing airfoil in translation motions; *s* is arc length, measured along the airfoil surface line from the cusp.

One can see that the *N*-scheme, as well as the $T^{0}$-scheme, provides close to the first order of accuracy. The $T^{1}$-scheme provides slightly higher than the first order of accuracy for a coarse surface mesh (for a number of panels less than 400) and also the first order of accuracy for finer meshes. The decrease of the order of accuracy in comparison to the 2nd order mentioned in [34], is caused by the BIE (4)–(6) solution behaviour: in the *x*-directed motion the exact solution $\gamma (\vec{\xi })$ is bounded and has the 1st kind discontinuity at the cusp, however, for the *y*-directed and rotational motions the exact solution has a weak singularity at the cusp point (Figure 2).

So, the values of the added masses $\lambda_{xx}$, $\lambda_{xy}$ and $\lambda_{x\omega }$ are estimated with the 2nd order of accuracy when using the $T^{1}$-scheme, Table 2.

Note that number of panels required for the relative error less than 1% and 0.1% for all the components of the added mass tensor differs significantly for the considered numerical schemes, Table 3.

It is clearly seen that the developed schemes, especially the $T^{1}$-scheme with piecewise-linear solution representation, provide much higher accuracy in comparison to the traditional DVM scheme.

If we consider another airfoil, i.e., an ellipse with semiaxes *a* and *b*, for which exact values of the added mass diagonal tensor components are known [41] and non-diagonal components are equal to zero,

$$\lambda_{xx}^{*}=\rho \pi b^{2},\;\lambda_{yy}^{*}=\rho \pi a^{2},\;\lambda_{\omega \omega }^{*}=\frac{1}{8}\rho \pi {\left(a^{2}-b^{2}\right)}^{2},$$

the opposite phenomenon is observed. In the general case, the *N*-scheme and the $T^{0}$-scheme provide the 1st order of accuracy for the BIE solution, while the $T^{1}$-scheme provides the 2nd order of accuracy. However, since the airfoil and its discretization are symmetric, and the solution in case of the added masses computation is also symmetric, all considered numerical schemes provide the 2nd order of accuracy, Table 4.

One can see that for the elliptical airfoil all the numerical schemes provide nearly the same accuracy. However, for arbitrary non-symmetric smooth airfoil, the $T^{1}$-scheme will be much more accurate in comparison to the others. Moreover, a more accurate $T^{2}$-scheme with piecewise-quadratic solution representation also can be used, but it requires airfoil surface line representation with curvilinear panels. Such a technique is described in [44,45]. The $T^{2}$-scheme is much more complicated in comparison to the $T^{1}$-scheme and it seems that its application in this problem is hardly can be justified. Note that the mentioned schemes $T^{0}$, $T^{1}$ and $T^{2}$ provide the 1st, 2nd and 3rd orders of accuracy in $L_{1}$-norm with respect to the solution of the BIEs (4)–(6).

## 6. Software Implementation of Vortex Methods

Until now, a small number of software implementations of vortex methods, preferably open source, are known, which would provide a possibility of solving a wide class of problems and would be available to researchers. Omega2D [46,47], as well as FlowVPM [48,49] projects can be pointed out that started in 2018 and 2019, however their capabilities, connected with modern mathematical models implementation and high-performance computing, remain at a rather modest level. The authors started in 2017 the VM2D project [50,51], where the viscous vortex domains (VVD) method is implemented together with developed *T*-schemes for numerical solving the BIE.

The source code of VM2D is written in C++ and has a modular structure in order to provide the possibility of its further development by introducing new mathematical models and algorithms, as well as its adaptation to new types of problems. It is cross-platform software and can be compiled under Windows and Linux by using MSVC, GCC, Clang or Intel C++ Compiler (as well as other compilers which support C++11 standard). External linear algebra library Eigen [52] is used in VM2D for standard operations with matrices and vectors.

OpenMP and MPI technologies are used for computations speedup on multi-core and multiprocessor cluster systems, and Nvidia CUDA technology is also supported. It is also possible to solve simultaneously (on multi-processor systems) a number of different tasks, which are organized automatically as a queue.

The results of parallelization efficiency investigation are presented in [53]. Here we note only that the most time-consuming operations in the vortex method numerical algorithm are convective and diffusive velocities computation of the vortex particles. Due to the possibility of their fully independent computation for different particles, the scalability of the algorithm on the whole is rather high, both for OpenMP and MPI technologies. Normally, the hybrid model of computations, namely MPI + OpenMP, on cluster systems is used, because each node of modern clusters is multiprocessor and multicore system with shared memory. However, the code execution on graphical accelerators (GPU) by using Nvidia CUDA technology seems to be the most efficient, its utilization makes it possible to speed up the computations dramatically [54]. The hybrid parallelization model, MPI + (OpenMP + Nvidia CUDA), is also supported, however in this case the MPI efficiency decreases: for a cluster system with four nodes, each equipped with GPU Tesla V100, the speedup value is slightly less than 3. At the same time, one should have in mind that the Tesla V100 accelerator provides speedup comparable to one that can be achieved at hundreds of CPU cores.

Programmer’s guide to VM2D is being generated automatically by using doxygen tool. It includes full information about all the classes implemented in VM2D: description of all the class members and methods. Relationships between the classes are shown in graphical mode, as well as execution diagrams of the functions. A html version is available online [55] and it is updated automatically via the Travis-CI service after every modification of source code and its push on GitHub.

**Figure 3:**
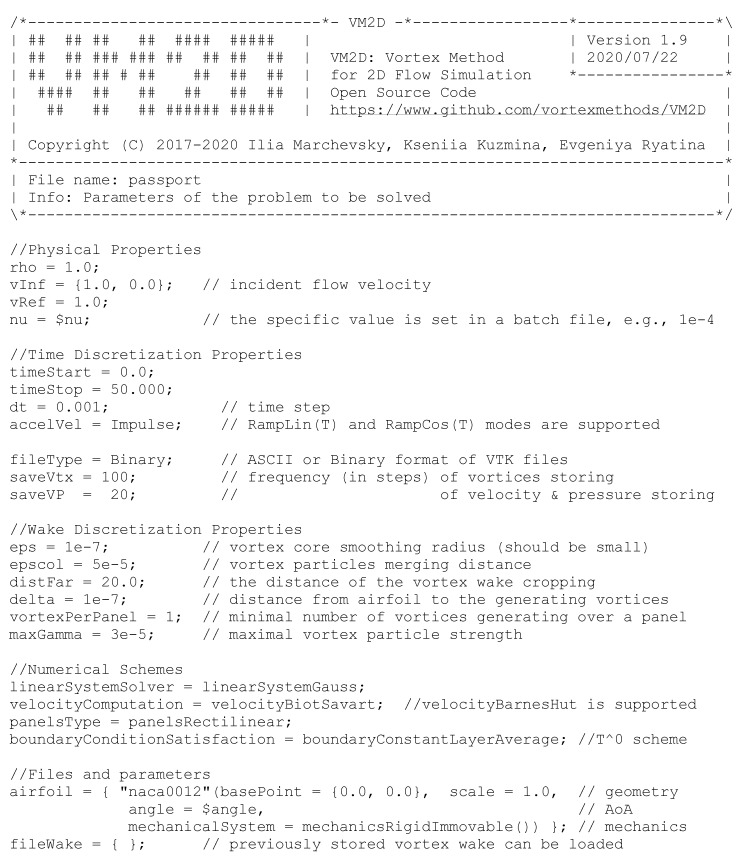
An example of the input file that describes the problem of flow simulation in VM2D.

All input data should be prepared in text files, where similar to C++ syntax (separators, comments, line-breaks, etc.) is used for general data representation and braces for list components representation. It is also possible to introduce some user-defined variables in order to simplify and automate somehow the solution of a series of similar problems that differ only by some parameters. A typical input file is shown in Figure 3.

The results of simulations are saved in text or binary VTK (Visualisation ToolKit [56]) format for physical fields, which values are known at nodes or cells of some spatial grid or at particles: velocity and pressure fields snapped on some grid, as well as vortex particles positions and their properties.

Tabular data computed at time steps such as hydrodynamic loads acting the airfoils, the airfoil positions and velocities, some time statistics, etc., are stored as simple text TXT or CSV (Comma-Separated Values) formats.

All the results of flow simulation presented in this paper, are obtained by using the VM2D code.

## 7. Proper Orthogonal Decomposition

The application of artificial intelligence and machine learning methods for CFD results processing has a rich history, described, in particular in review paper [57]. Various machine learning methods can be used to compress and store data—the most common are Proper Orthogonal Decomposition (POD), also known as principal component analysis (PCA), and Dynamic Mode Decomposition (DMD).

Both POD and DMD are modal decomposition approaches that allow for reducing the amount of stored experimental or numerical data by its low-dimensional (or low-rank) approximate representations with high enough accuracy. The main idea of these methods is to identify the coherent modes that dominate in the measured signal. The DMD can be efficiently used when it is necessary to reveal frequency information and corresponding spatial structures throughout the field [58,59,60]. So, the DMD seems to be preferable for a detailed investigation of the flow structure. In this research, the POD technique is used for data processing and compression, since normally it requires a smaller number of modes in comparison to DMD [58] for data reconstruction with a given accuracy. In this research, the main purpose of the model order reduction technique usage is to store the results of numerical simulation as efficiently as possible, having in mind its further processing or usage in other applications. Thus, the possibility of the flow structure investigation by using the POD technique here is considered only as an additional feature, which is however rather valuable.

It is well-known that the proper orthogonal decomposition (POD) method, makes it possible to extract the flow features from experimental or numerical data and construct low-dimensional representations for initially high-dimensional data. Note that the application of the POD technique can also be considered as some filtering tool, which permits to discard non-essential small-scale features of the numerical solution. The scope of the POD is very broad in modern applications, such as micromechanics [61,62], aeroelasticity [63,64], fluid mechanics [65,66], aeroacoustics [67], and others.

In this regard, it is promising to use POD together with vortex methods.

The POD technique was introduced in 1967 [68] as an attempt to decompose a random vector field of turbulent fluid motion as a set of deterministic functions, each of which captures some part of the total oscillating kinetic energy in the flow. In recent decades, the study of turbulent or extended chaotic systems has made significant progress. Two main achievements can be distinguished: first, the recognition of the existence and high significance of coherent structures (defined as strongly stable space-time structures in the dynamic evolution of a system) in weakly and even strongly chaotic systems, and, second, the adoption of mathematical methods that came from studies of nonlinear dynamical systems [69], which led to the need to improve data storage and processing. Thus, POD has become one of the classic tools of experimental and numerical hydrodynamics. The main task of this method is to divide the data set into orthogonal spatial and temporal modes, which allows one to study the data set most efficiently.

The essence of the method is as follows: the considered physical field that depends on spatial coordinates and time, is approximately replaced by a sum of the spatial modes ${\left\{\varphi_{k}^{\left(h\right)}\left(\vec{r}\right)\right\}}_{k=1}^{m}$ with time-dependent coefficients:

$$\Psi^{\left(h\right)}\left(\vec{r},\;t\right)\approx \sum_{k=1}^{m}a_{k}\left(t\right)\varphi_{k}^{\left(h\right)}\left(\vec{r}\right),$$

where the upper index (h) means that the corresponding field is a grid function. The desired property of such an approximation is a small number *m* of required basis functions compared to the number of time steps together with the high accuracy of the field $\Psi^{\left(h\right)}$ reconstruction.

The condition of orthogonality of the basis in the sense of a *n*-dimensional space, where *n* is the number of grid nodes or cells, is also desirable (the values of $\Psi^{\left(h\right)}$ at a chosen time moment in the nodes/cells of the grid are considered as the elements of an *n*-dimensional vector). The latter requirement, among other things, significantly simplifies the procedure for calculating the coefficients $a_{k}\left(t\right)$.

Let us assume that there is a certain amount of computed data set in the grid nodes at consecutive times $t_{1},\;t_{2},\;\ldots ,\;t_{N}$. It is assumed that this data corresponds to the results of some unsteady CFD simulation stored with a certain step (usually significantly exceeding the time step in the simulation). When studying the scalar field, i.e., pressure field, we consider the initial data as a set of *n*-dimensional vectors ${\left\{p^{\left(h\right)}\left(t_{i}\right)\right\}}_{i=1}^{N}$. For *d*-dimensional vector field processing, such as velocity field, vectors ${\left\{{\vec{v}}^{\left(h\right)}\left(t_{i}\right)\right\}}_{i=1}^{N}$ are represented as (d·n)-dimensional vectors in which all the components of the solution in all cells are written in a row. So hereinafter we will not distinguish scalar and vector fields and denote the data from initial snapshots as ${\left\{x^{\left(h\right)}\left(t_{i}\right)\right\}}_{i=1}^{N}$.

To build a basis ${\left\{\varphi_{k}^{\left(h\right)}\right\}}_{k=1}^{N}$, the following operations are performed.

1. The calculation of the symmetric covariance matrix *R* of N×N size with components
  $$R_{ij}=\frac{1}{N}\left(x^{\left(h\right)}\left(t_{i}\right),\;x^{\left(h\right)}\left(t_{j}\right)\right),$$
  where (u,v) means scalar product of the vectors *u* and *v*.
2. The calculation of eigenvalues ${\left\{\lambda_{k}\right\}}_{k=1}^{N}$ and eigenvectors ${\left\{\nu_{k}\right\}}_{k=1}^{N}$ for the matrix *R*; the dimension of the vectors $\nu_{k}$ is equal to number of snapshots *N*.
3. Setting the basis vectors, which determine spatial modes, as
  $$\varphi_{k}^{\left(h\right)}\left(\vec{r}\right)=\sum_{j=1}^{N}{\left(\nu_{k}\right)}_{j}x^{\left(h\right)}\left(t_{j}\right),$$
  where ${\left(\nu_{k}\right)}_{j}$ denotes the *j*-th component of the *k*-th eigenvector.
4. Calculating the time-dependent coefficients ${\left\{a_{k}\left(t_{i}\right)\right\}}_{k=1}^{N}$ that allow for solution reconstructing as a linear combinations of spatial modes at all time steps *i*:
  $$a_{k}\left(t_{i}\right)=\frac{\left(x^{\left(h\right)}\left(t_{i}\right),\;\varphi_{k}^{\left(h\right)}\right)}{\left(\varphi_{k}^{\left(h\right)},\;\varphi_{k}^{\left(h\right)}\right)}=\frac{\left(x^{\left(h\right)}\left(t_{i}\right),\;\varphi_{k}^{\left(h\right)}\right)}{{∥\varphi_{k}^{\left(h\right)}∥}^{2}}.$$
5. Representation of the solution at the *i*-th time step:
  $$x^{\left(h\right)}\left(t_{i}\right)=\sum_{k=1}^{N}a_{k}\left(t_{i}\right)\varphi_{k}^{\left(h\right)}.$$

Note that the following optimal property of the POD basis is satisfied [69]:

- For arbitrary *n*-dimensional vector $\psi^{\left(h\right)}$ such as $∥\psi^{\left(h\right)}∥=∥\varphi_{1}^{\left(h\right)}∥$,
  $$\sum_{i=1}^{N}{\left(x^{\left(h\right)}\left(t_{i}\right),\;\varphi_{1}^{\left(h\right)}\right)}^{2}\geq \sum_{i=1}^{N}{\left(x^{\left(h\right)}\left(t_{i}\right),\;\psi^{\left(h\right)}\right)}^{2};$$
- For arbitrary *n*-dimensional vector $\psi^{\left(h\right)}$ such as $∥\psi^{\left(h\right)}∥=∥\varphi_{2}^{\left(h\right)}∥$ and $\left(\psi^{\left(h\right)},\;\varphi_{1}^{\left(h\right)}\right)=0$,
  $$\sum_{i=1}^{N}{\left(x^{\left(h\right)}\left(t_{i}\right),\;\varphi_{2}^{\left(h\right)}\right)}^{2}\geq \sum_{i=1}^{N}{\left(x^{\left(h\right)}\left(t_{i}\right),\;\psi^{\left(h\right)}\right)}^{2};$$
  and so on.

Due to these inequalities, the following property is satisfied: for any value of the number m=1,…,N of basis modes, the sum of the squares of the approximation errors

$$\sum_{i=1}^{N}{∥x^{\left(h\right)}\left(t_{i}\right)-\sum_{k=1}^{m}a_{k}\left(t_{i}\right)\varphi_{k}^{\left(h\right)}∥}^{2}$$

takes the smallest value for all possible ways of choosing orthogonal basis modes $\left\{\varphi_{k}^{\left(h\right)}\right\}$, k=1,…,m. This means that each subsequent basis mode $\varphi_{k}^{\left(h\right)}$ is chosen so as to hold the maximum possible amount of “information” about the original solution that also can be treated as the “energy” which is contained in the solution.

In this paper, the POD technique is applied to velocity and pressure fields ${\vec{v}}^{\left(h\right)}\left(\vec{r},\;t\right)$ and $p^{\left(h\right)}\left(\vec{r},\;t\right)$ processing. The relative error of the field $x^{\left(h\right)}\left(\vec{r},\;t\right)$ approximation with *m* modes obtained by using the POD technique at the particular time step $t_{i}$ is calculated as follows

$$\varepsilon^{\left(m\right)}\left(t_{i}\right)=\frac{{\left(\int_{\Omega }{∥x^{\left(h\right)}\left(\vec{r},\;t_{i}\right)-x_{m}^{\left(h\right)}\left(\vec{r},\;t_{i}\right)∥}^{2}d\Omega \right)}^{1/2}}{{\left(\int_{\Omega }{∥x^{\left(h\right)}\left(\vec{r},\;t_{i}\right)∥}^{2}d\Omega \right)}^{1/2}},$$

where the fields $x^{\left(h\right)}\left(\vec{r},\;t_{i}\right)$ and $x_{m}^{\left(h\right)}\left(\vec{r},\;t_{i}\right)$ mean the initial grid function and its approximate representation

$$x_{m}^{\left(h\right)}\left(\vec{r},\;t_{i}\right)=\sum_{k=1}^{m}a_{k}\left(t_{i}\right)\varphi_{k}^{\left(h\right)},$$

and the integrals are understood as the corresponding quadrature sums.

The value that expresses the error on all the time steps, is calculated as follows

$$\varepsilon^{\left(m\right)}=\frac{{\left(\int_{t_{1}}^{t_{N}}dt\int_{\Omega }{∥x^{\left(h\right)}\left(\vec{r},\;t_{i}\right)-x_{m}^{\left(h\right)}\left(\vec{r},\;t_{i}\right)∥}^{2}d\Omega \right)}^{1/2}}{{\left(\int_{t_{1}}^{t_{N}}dt\int_{\Omega }{∥x^{\left(h\right)}\left(\vec{r},\;t_{i}\right)∥}^{2}d\Omega \right)}^{1/2}}.$$

Note that the values $\varepsilon^{\left(m\right)}$ coincide with the square root of the sums of the corresponding normalized eigenvalues:

(8) $$\delta^{\left(m\right)}=\sqrt{\sum_{k=m+1}^{N}\lambda_{k}/\sum_{k=1}^{N}\lambda_{k}}.$$

This property of the POD algorithm makes it possible to estimate the reconstruction error easily.

## 8. Numerical Experiments

Let us consider a set of model problems connected to flow simulation around some airfoil. Our main aim is to analyze the possibility of the vortex methods application to correct reconstruction of the flow structure in the neighborhood of the airfoil, which determines hydrodynamical loads acting the airfoil. The quality of flow simulation in far-field is not discussed in this paper, however, in some engineering applications, e.g., at flow simulation around tandems of airfoils [70], it also can be important.

### 8.1. The Blasius Problem

The Blasius flow is one of the simplest and the most popular test that allows for checking if the viscous stresses in the flow are modelled correctly or not.

A steady two-dimensional laminar boundary layer is simulated that is formed on a semi-infinite plate which is parallel to a steady incident flow. The analytical asymptotic solution is known for this flow [39,71]. In numerical simulation a thin plate of unit length L=1.0 and width H=0.004 with semicircular tips was considered; the incident flow has unit velocity $V_{\infty }=1$, its density ρ=1 and kinematic viscosity $\nu =10^{-3}$. We assume hereinafter that all the values are dimensionless.

The airfoil was discretized into 5088 panels of the same length, parameter $\Gamma_{\max}$ that determines maximal strength of vortex particles was chosen equal to $4\times 10^{-5}$, time step for unsteady flow simulation $\Delta t=2\times 10^{-4}$.

**Figure 4:**
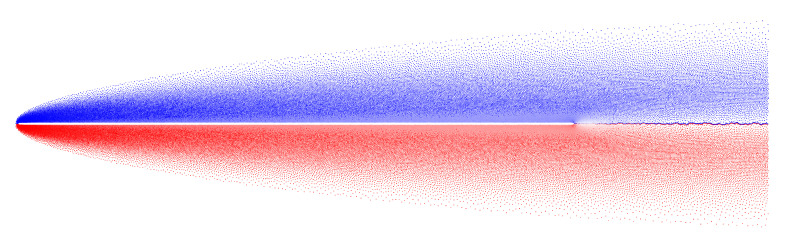
Vortex particles positions in steady-state regime for the Blasius flow.

The positions of vortex particles at time moment t=7.5 are shown in Figure 4.

**Figure 5:**
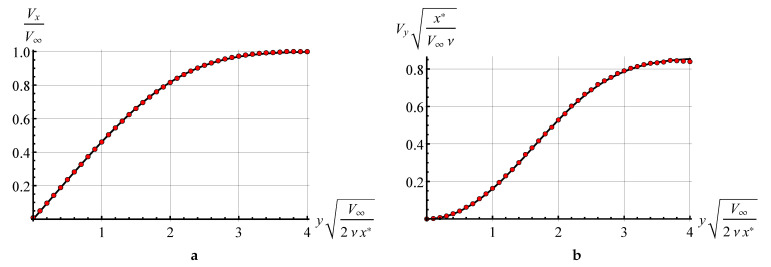
Horizontal (**a**) and vertical (**b**) dimensionless velocity profiles in cross-section $x_{*}=0.25$ (red circles), in comparison to analytical solution (black solid line).

Velocity profiles for horizontal and vertical velocity components were measured in the cross-section at $x_{*}=0.25$. Their comparison to analytical solution is shown in Figure 5.

It is clearly seen that the vortex particle method implementation, based on viscous vortex domain method, allows for very accurate simulation in laminar viscous boundary layers.

### 8.2. The Flow in the Channel with Backward Facing Step

**Figure 6:**
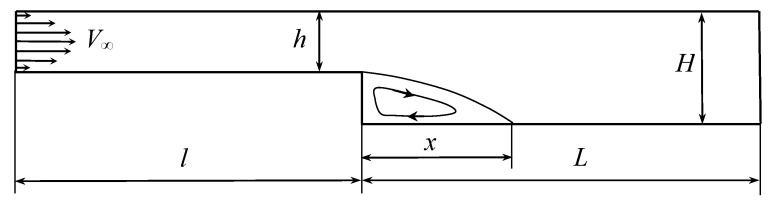
Scheme of the channel with backward-facing step.

Another model problem widely used for numerical methods verification is internal flow simulation in the channel with a backward-facing step. In [72], the results of experiments and numerical simulations are shown for this problem. The shape of the channel is shown in Figure 6. In numerical simulation the height of the input and output sections were chosen as h=1 and H=1.94, similar to [72]. The velocity profile in the inlet cross-section is assumed to be parabolic, which corresponds to the Poiseuille flow. The position of the reattachment point *x* is estimated for different values of the Reynolds number.

In order to eliminate the influence of the inlet and outlet boundaries, simulations were performed for channels with different values of *l* and *L*; the lengths l=7 and L=13 are quite enough to simulate the flow in infinite channel. The increase of these values practically does not affect the reattachment point position.

The Reynolds number is defined in this problem as

Re=VDν,

where *V* is the average flow velocity in the input section equal to two-thirds of maximal inlet velocity, *D* is the hydraulic diameter of the inlet section equal to twice its height, D=2h, and ν is the kinematic viscosity coefficient.

The channel perimeter was discretized into 1400 panels of the same length, parameter $\Gamma_{\max}=10^{-3}$, time step for flow simulation $\Delta t=10^{-2}$. The velocity profile in the inlet cross-section was simulated as the summary influence of 100 point sources, which strengths were chosen in such a way to reconstruct the Poiseuille profile with maximal velocity V=1. Kinematic viscosity is variable and corresponds to the Reynolds number values in the range from 50 to 400.

**Figure 7:**
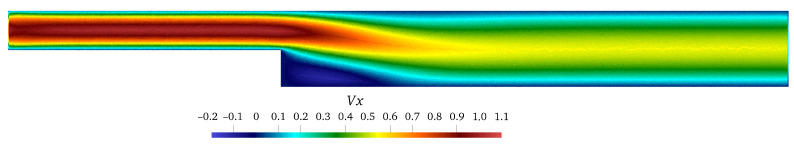
Horizontal component of the flow velocity in the channel with backward-facing step.

**Figure 8:**
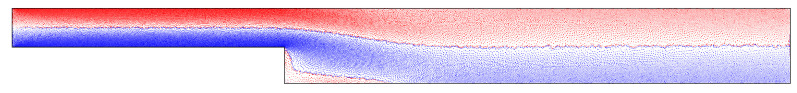
Vortex particles positions in steady-state regime for the flow in the channel with backward-facing step.

The horizontal velocity distribution in steady-state regime at time moment $t_{*}=200$ for Re=100 is shown in Figure 7 and the positions of vortex particles at the same time moment are shown in Figure 8.

**Figure 9:**
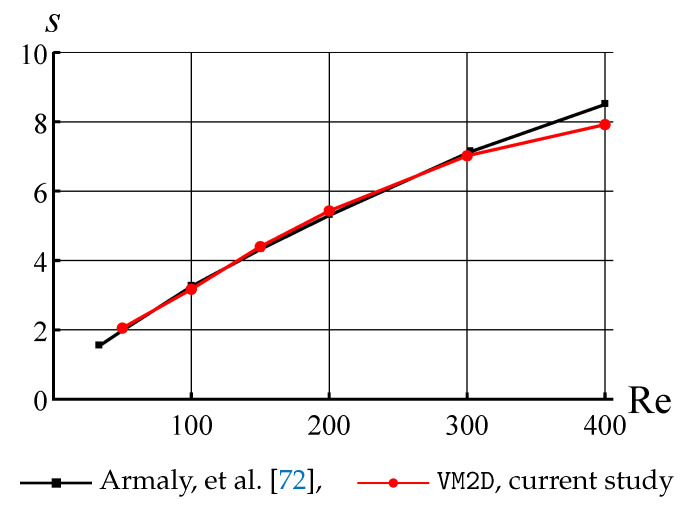
Positions of the flow reattachment point *s* for different Reynolds numbers.

The positions of the flow reattachment point obtained in simulations in VM2D were compared to experimental data [72] for the Reynolds number in the range 50…400. Figure 9 shows the dependency of the value s=x/(H−h) against the Reynolds number.

It is seen that the results are in good agreement in the range Re=50…300. The significant difference between the results occurs at Re>300, apparently due to the fact, also noted in [72], that the real flow becomes essentially three-dimensional, so two-dimensional simulation is incorrect.

So, the following conclusion should be done: in the general case, two-dimensional flow simulation remains correct only for the regimes that are stable in real flows, when long cylinders are considered in the cross flow. However, for these cases, vortex methods show a rather high accuracy.

### 8.3. Flow Separation Angle for a Circular Cylinder

If the airfoil surface line is smooth enough and there are no sharp edges and corner points, the position of the separation point depends significantly on the Reynolds number. The positions of separation point, obtained as a result of numerical simulation, say a lot about the correctness of flow regime simulation in the whole and, in particular, about the accuracy of the hydrodynamic loads computation, because, for example, the drag force value is strongly connected to the separation point position.

We consider only flows at rather low Reynolds numbers, at it follows from the previous results, namely a flow around a circular cylinder at Re=20…200. In this range, two flow regimes can be distinguished: steady regime, when the flow after the airfoil is symmetric, and the regime with vortex shedding and von Karman vortex street formation. For symmetrical regimes (the Reynolds numbers up to 40) the separation point is practically immovable, while in modes with vortex shedding it moves periodically along some arc during the vortex-shedding period.

**Figure 10:**
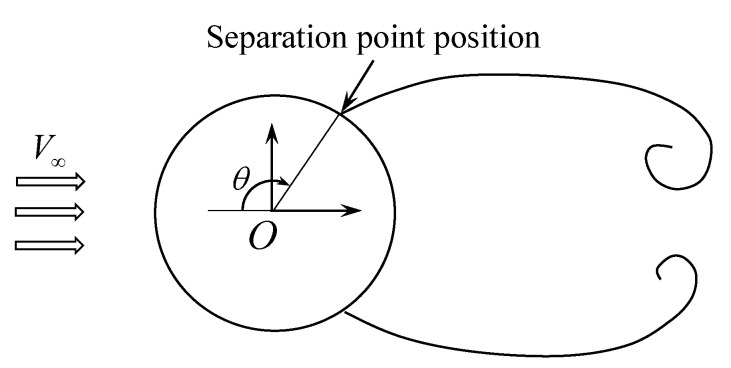
The position of the separation point for the circular airfoil in cross flow.

The separation angle value θ is measured as shown in Figure 10.

Numerical simulations were performed for the circular airfoil of unit diameter discretized into 1000 panels of equal length, incident flow velocity $V_{\infty }=1$, maximal vortex particle strength $\Gamma_{\max}=2\times 10^{-4}$, time step $\Delta t=2\times 10^{-3}$.

**Figure 11:**
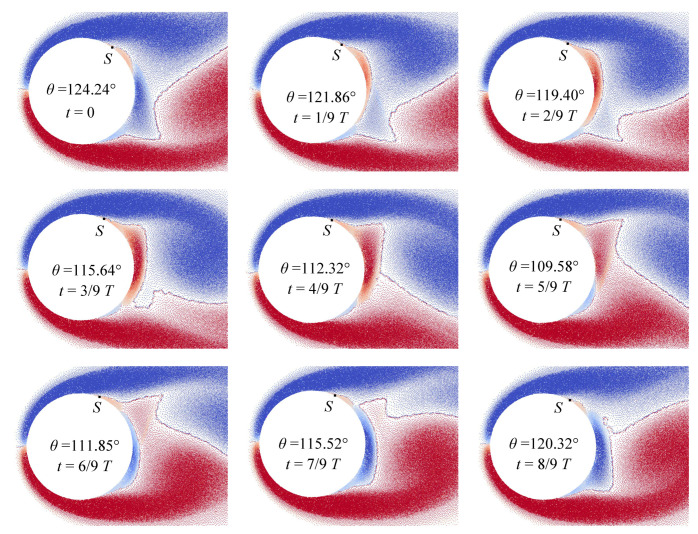
Vortex particles positions around the airfoil and the positions of separation points for different time instants at Re=200.

Figure 11 shows the vortex particles distribution in the near-wall region around the airfoil and the positions of separation point for different time instants during one vortex-shedding period *T* for the case of flow simulation at Re=200.

**Figure 12:**
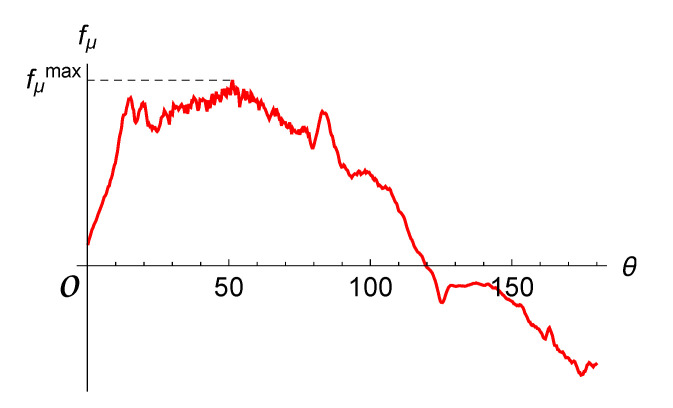
Distribution of the share stresses along the airfoil surface line for the top part of the circular airfoil.

The angular position of the separation point corresponds to the point at which the shear stress vanishes. For example, the distribution of the share stresses along the airfoil surface line for Re=200 at time $t_{*}=2/9T$ is shown in Figure 12. It is seen that the instant value for the separation angle $\theta \approx 120^{\circ }$.

The Table 5 shows the obtained values for the separation angle at different Reynolds number. For the steady symmetrical regimes, only one value for the separation angle $\bar{\theta }$ is given, which varies just slightly in time during the numerical simulation. For the quasi-steady regimes with von Karman vortex street formation the position of the separation point varies significantly during the vortex shedding period, the average value $\bar{\theta }$ for the separation angle is given, as well as the range $\theta_{min}\ldots \theta_{max}$ in which θ changes periodically.

**Figure 13:**
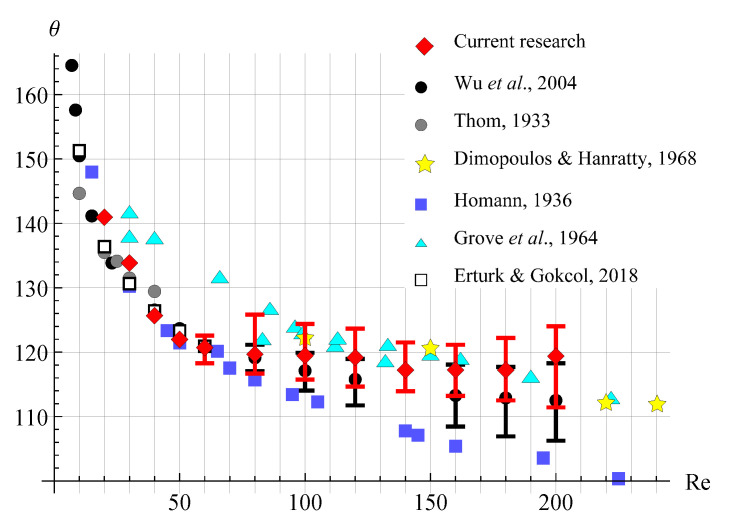
The separation point angular position against the Reynolds number obtained in current research in comparison to the results of other authors.

A detailed review of the results obtained numerically and experimentally by different authors for the considered problem is given in [73]. Figure 13 shows a comparison of the results obtained in current study from numerical simulation using VM2D code, with experimental and numerical results of other authors: experimental investigations by Thom [74], Dimopoulos and Hanratty [75], Homann [76], Grove, et al. [77], and numerical investigations by Wu, et al. [73] and Erturk and Gokcol [78]. Results in [78] are given only for the Reynolds numbers up to 60 since the authors consider only stationary modes.

It is seen from Figure 13 that the results obtained in the current study for Reynolds numbers up to 140 are in good agreement with the results of other researchers. While for the Reynolds number from 160 to 200, the separation angle seems to be slightly higher than the results of numerical simulations of recent years, however, the average values agree quite well with the results obtained experimentally in [75,77].

Thus, the vortex method usage makes it possible to simulate flow separation on a smooth surface at low Reynolds numbers (up to 140 for circular cylinder).

### 8.4. The POD Technique Application to Data Compression and Flow Regimes Analysis

The number of vortex particles in the flow domain in the previously discussed problem of flow around circular cylinder simulation has an approximately quadratic dependence against the number of panels on the airfoil surface line. This is due to the fact that the minimal distance between vortex particles is usually chosen proportionally to the length of the panel (vortex particles that are closer to each other are merged into one vortex if its summary strength is lower than $\Gamma_{\max}$).

A typical number of vortex elements in the above discussed numerical simulations have the order of 300,000. In principally, the velocity field can be reconstructed at an arbitrary point by using the Biot–Savart law, however, it requires storing positions and strength of all the vortex particles for each snapshot. It is easy to estimate that one such “snapshot” has approximately 7 Mb size, while the number of snapshots has the order of 1000. So, all the raw data takes approximately 5…10 Gb. Note that velocity reconstruction in such an approach requires significant computational resources.

If one computes velocity and pressure at the nodes of a rather fine spatial mesh that contains approximately 60,000 points, such a snapshot would have 1.5 Mb size, and total data would take about 1…2 Gb.

However, if such velocity-pressure snapshots are used for the POD expansion construction, each mode stored in a file has, again, approximately 1.5 Mb in size.

Now we consider in details the results of flow simulation around the circular cylinder at Re=200 in quasi-steady regime (from $t_{*}=80$ to $t_{**}=160$); all other parameters of simulation are the same as in the previous section.

**Figure 14:**
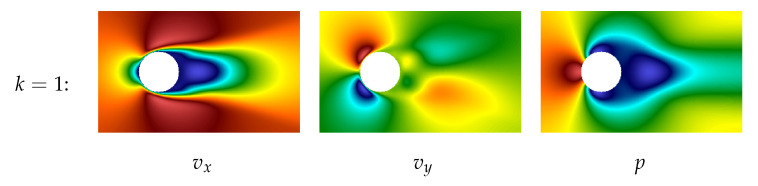
The first POD-mode for the flow simulation around the circular cylinder at Re=200.

The plot of the first POD-mode for both components of the velocity field and pressure field are shown in Figure 14. This mode describes precisely the averaged characteristics of the flow. Note that hereinafter the POD technique is applied to vector field $\vec{v}$ (together to both components) and separately to pressure field *p*.

**Figure 15:**
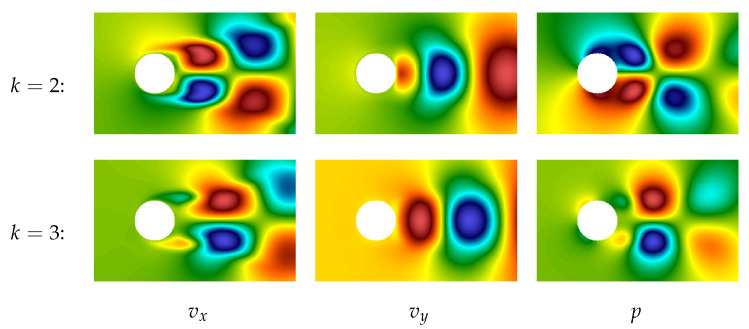
The 2nd and 3rd POD-modes for the flow simulation around the circular cylinder at Re=200.

The second and the third POD-modes are very similar, their plots are shown in Figure 15. One can notice that the 2nd and the 3rd modes are “phase-shifted” in some sense. It is quite natural because these modes correspond to large vortex structures (von Karman vortices), and their linear combination allows for describing the vortices running from left to right.

**Figure 16:**
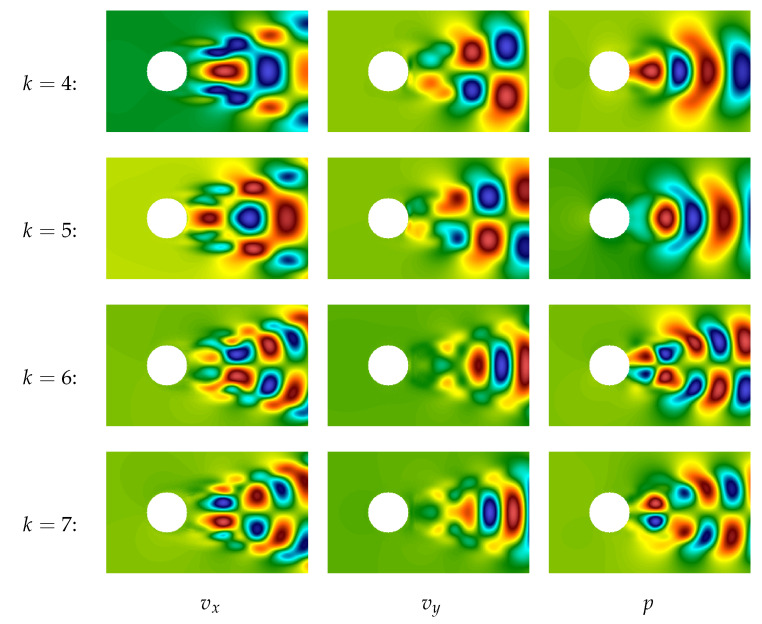
The POD-modes from 4th to 7th for the flow simulation around the circular cylinder at Re=200.

Plots of the next four modes (from 4th to 7th) are shown in Figure 16. They correspond to smaller-scale vortex structures and again are “phase-shifted” (the 4th in comparison to the 5th and the 6th in comparison to the 7th), so they also describe running vortices.

**Figure 17:**
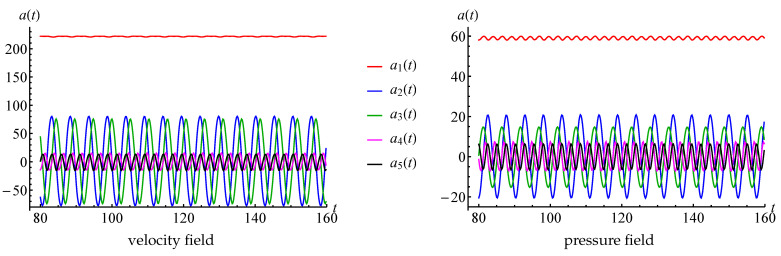
Time coefficient dependencies $a_{k}\left(t\right)$ for first five POD-modes for the flow simulation around circular airfoil at Re=200.

The thoughts about simulation of running vortices are in good agreement with the values of time coefficients $a_{k}\left(t\right)$ that correspond to the first five POD-modes (coefficients $a_{6}\left(t\right)$ and $a_{7}\left(t\right)$ are very similar to $a_{4}\left(t\right)$ and $a_{5}\left(t\right)$ and are not shown in the plot), Figure 17. One can see that $a_{1}\left(t\right)$ is very close to constant, $a_{2}\left(t\right)$ and $a_{3}\left(t\right)$ are periodical with zero average value, and phase shifted, and the same for $a_{4}\left(t\right)$ and $a_{5}\left(t\right)$.

**Figure 18:**
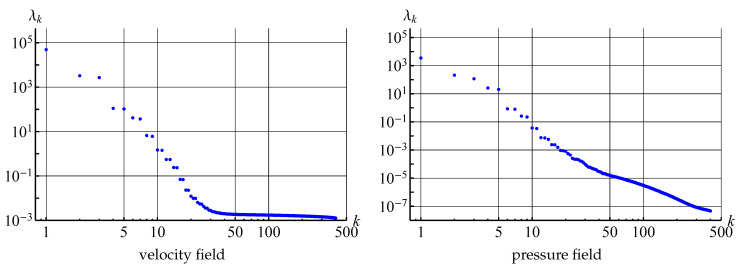
Eigenvalues $\lambda_{1}\ldots \lambda_{400}$ of the covariance matrix (in logarithmic scale) for the unsteady flow simulation around circular cylinder at Re=200.

The plot of eigenvalues of the covariance matrices for velocity (considered as vector field) and pressure fields that are processed in the POD algorithm are shown in Figure 18. It is seen that the eigenvalues decrease rapidly. The maximal eigenvalue corresponds to the first mode (averaged flow), $\lambda_{2}\approx \lambda_{3}$ corresponds to the modes that describe large-scale von Karman running vortices, $\lambda_{4}\approx \lambda_{5}$ and $\lambda_{6}\approx \lambda_{7}$ correspond to smaller-scale running vortices. Six more pairs of close eigenvalues are clearly observable, which also describes smaller-scale vortices. Some modes with numbers higher than 19 also correspond to some small-scale vortex structures in the flow domain, some others do not have physical meaning and seem to be some type of noise, maybe connected with the properties of the numerical method. In any case, the values of the eigenvalues with higher numbers are many times smaller in comparison to the first eigenvalues.

In Table 6, the errors of velocity and pressure fields reconstruction are shown computed according to (8) for the different number of the POD-modes taken into account at the fields reconstruction.

It is seen that the errors of both fields reconstruction decrease at nearly the same rate, and only nine first POD-modes storing allows for very precise solution reconstruction (with 1% error) during all the time period in quasi-steady regime. Note that the POD-modes analysis hardly makes it possible to determine the position of the separation point on the cylinder surface line, but it allows to reduce dramatically the size of data that should be stored in order to have the possibility for solution reconstruction.

### 8.5. Flow around a Wing Airfoil Simulation

In previous subsections, it was shown that two-dimensional vortex methods allow for correct simulation for airfoils with smooth surface lines only for low-Reynolds flows. However, if we consider wing airfoils at a rather high Reynolds number, the obtained results for drag and lift forces are in acceptable agreement with experimental data.

**Figure 19:**
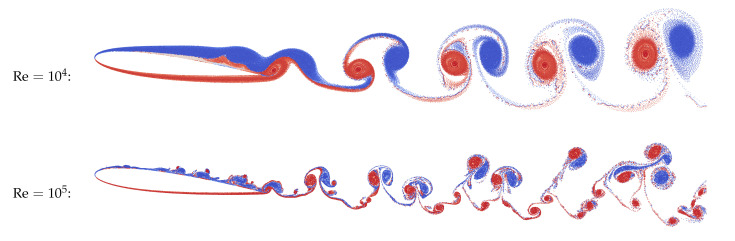
Vortex wake after the NACA-0012 wing airfoil for AoA $\alpha =6^{\circ }$ at Re=104 and Re=105.

Vortex wakes after NACA-0012 airfoil in quasi-steady regime are shown in Figure 19 for the Reynolds numbers Re=104 and Re=105; angle of incidence $\alpha =6^{\circ }$. Parameters of the flow simulation in VM2D were the following: wing airfoil with unit chord at unit incident flow was considered, the airfoil surface line was discretized into 1000 panels, maximal vortex particle strength $\Gamma_{\max}=3\times 10^{-5}$, time step $\Delta t=10^{-3}$ (input file for this case was shown in Figure 3).

**Figure 20:**
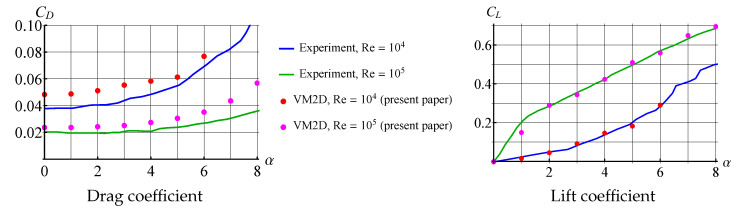
Drag force and lift force coefficients against the AoA for NACA-0012 airfoil at Re=104 and Re=105.

Drag and lift coefficients for NACA-0012 airfoil, obtained experimentally by Winslow, et al. [79] and numerically in the VM2D code, are shown in Figure 20 for the Reynolds number values Re=104 and Re=105.

Despite the fact that the drag force coefficient is overestimated in numerical simulations, the main result of this simulation is the following: two-dimensional simulation permits to obtain correct results at rather high Reynolds numbers, if the airfoil with a sharp edge (or angle point) is considered, and the flow separation takes place at this point; the viscous vortex domains method allows for correct resolving the viscosity effect that is especially clearly seen for the lift coefficient dependency against the angle of attack (AoA).

Note that for AoA higher than shown in Figure 20, the results differ significantly from the experimental. This is due to the fact that after some critical AoA, the intensive flow separation takes place from the smooth part of the wing airfoil. This phenomenon hardly can be described and simulated correctly in the framework of “pure” two-dimensional simulation without any additional models, such as turbulence closure models. Recall that nowadays there are no such models developed for vortex methods, and its creation can be treated as a significant achievement in vortex methods development.

### 8.6. Unsteady Flow Simulation around Circular Cylinders

It seems that unsteady and transient regimes simulation is the most attractive area where Lagrangian vortex methods can be efficiently applied. As it was mentioned in Introduction, in number of engineering applications it is important to determine unsteady hydrodynamic loads, acting on the structure at transient regime. So another test case was considered that is connected with flow simulation around impulsively started cylinder.

**Figure 21:**
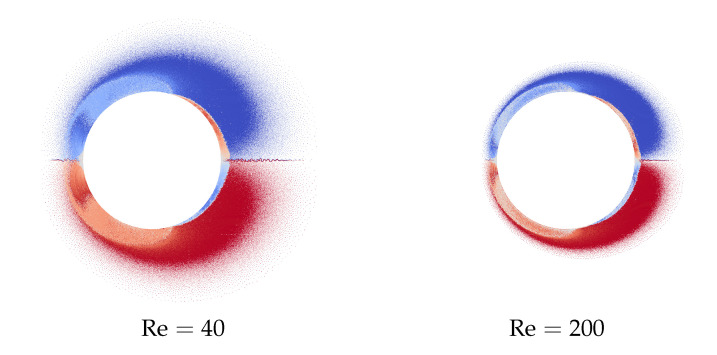
Vortex particles positions in vortex wakes after the impulsively started circular cylinder at Re=40 and Re=200 at time moment $t_{*}=0.5$.

Unsteady drag force computation for an impulsively started cylinder at different Reynolds number is also a “standard” test problem that have been investigated by many researchers. In Figure 21, the vortex wakes are shown for a circular cylinder of unit diameter that was discretized into 3200 panels of equal length. The media was considered to be still (zero incident velocity), while the airfoil starts to move impulsively from left to right with unit velocity. The value of $\Gamma_{\max}$ was chosen equal to $2\times 10^{-5}$, time step for flow simulation $\Delta t=10^{-4}$. Two cases were considered, for which media viscosity coefficients have been chosen in such a way to provide the Reynolds numbers Re=40 and Re=200.

**Figure 22:**
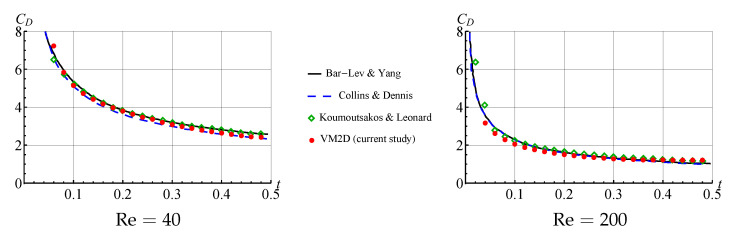
Unsteady drag force coefficient acting on the impulsively started circular cylinder at Re=40 and Re=200.

Time dependencies for summary drag force (caused by pressure distribution and viscous friction) for the considered cases are shown in Figure 22 in comparison to the results, obtained by Bar-Lev and Yang [80], Collins and Dennis [81] and Koumoutsakos and Leonard [82].

It is seen that the results of flow simulation are in good agreement with the results obtained by other researchers.

**Figure 23:**
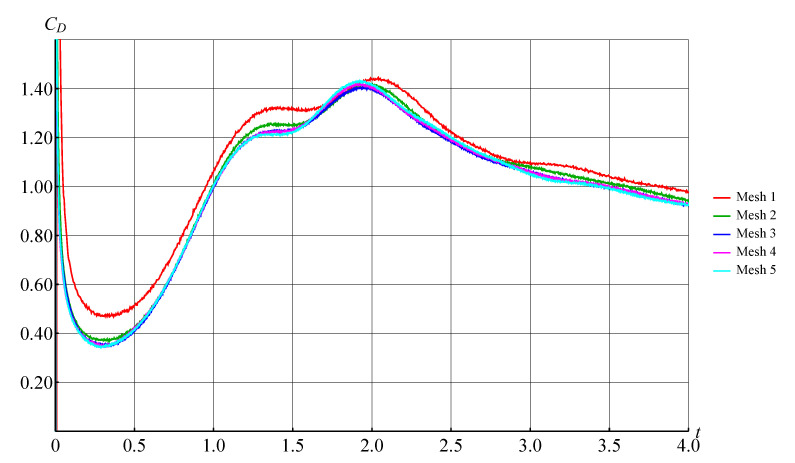
Results of mesh convergence investigation for unsteady drag force coefficient acting the impulsively started circular cylinder at Re=3000.

Similar simulation have been performed for higher Reynolds number, namely Re=3000, but the final time of simulation now was chosen ten times larger, equal to $t_{*}=5.0$. The results of numerical simulation of such flow for different discretization parameters are shown in Figure 23 for the unsteady drag force coefficient. These results can be considered as mesh convergence investigation. For simulations marked as “Mesh 1” …“Mesh 5”, the following parameters have been varied: maximal vortex particle strength $\Gamma_{\max}$ and time step Δt that were divided in half for each subsequent “Mesh”. Note that number of panels that approximate the airfoil surface line, was the same for “Meshes 1, 2, 3”, and then it was doubled twice for “Mesh 4” and “Mesh 5”.

It is clearly seen that the results of simulation for the set of parameters, marked as “Mesh 3, 4, 5” are close to each other. The finest parameters that correspond to the “Mesh 5” are the following: 3200 panels on the airfoil surface line, $\Gamma_{\max}=10^{-5}$, $\Delta t=2.5\times 10^{-4}$.

**Figure 24:**
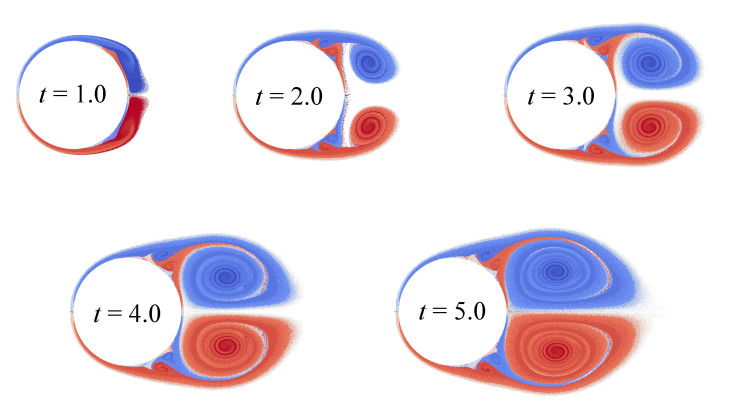
Vortex particles positions in vortex wakes after the impulsively started circular cylinder at Re=3000 at different time moments.

Vortex wakes at time moments t=1.0, t=2.0, t=3.0, t=4.0 and t=5.0 are shown in Figure 24.

**Figure 25:**
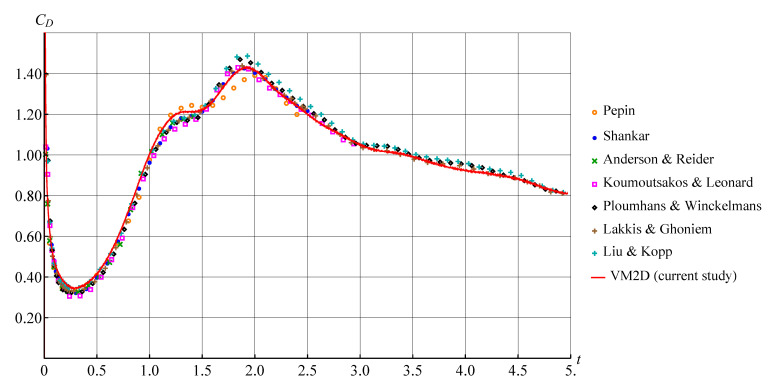
Unsteady drag force coefficient acting the impulsively started circular cylinder at Re=3000.

In [10,80,82,83,84,85,86,87,88,89,90,91,92,93,94,95,96] the results are presented for this problem at Re=3000. In Figure 25, the results obtained by using the VM2D code with the above mentioned discretization parameters set “Mesh 5” are shown in comparison to the results of Pepin [86], Shankar [87], Anderson and Reider [88], Koumoutsakos and Leonard [82], Ploumhans and Winckelmans [90], Lakkis and Ghoneim [93] and Liu and Kopp [95] thatseem to be the most accurate.

A very good agreement is observed; it should be pointed out that the results of computations using the VM2D code that are shown in Figure 25 by red color, are just unsteady results, presented without any averaging. So, the vortex method, based on the developed *T*-schemes, makes it possible to obtain non-oscillating unsteady hydrodynamic loads.

Note that in the last simulation number of vortex particles has order of 1 million, and storing of their positions and strength for all the snapshots requires a lot of disk space (each snapshot file size is about 20–30 Mb).

**Figure 26:**
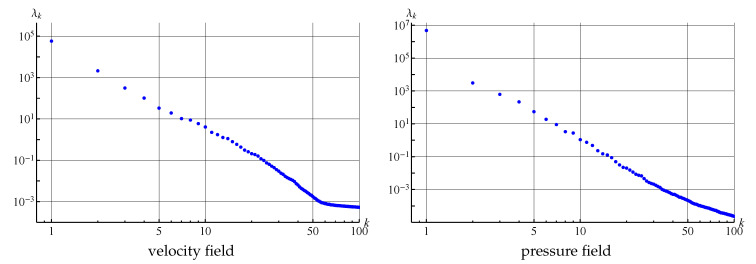
Eigenvalues $\lambda_{1}\ldots \lambda_{100}$ of the covariance matrix (in logarithmic scale) for the unsteady flow simulation around impulsively started circular cylinder at Re=3000.

At the same time if 1000 snapshots are stored for the last simulation (with time step 0.005), the POD technique can be efficiently applied to this data. The first 100 eigenvalues of the covariance matrices for velocity (considered as vector field) and pressure fields are shown in Figure 26, it is seen that they decrease rather rapidly.

**Figure 27:**
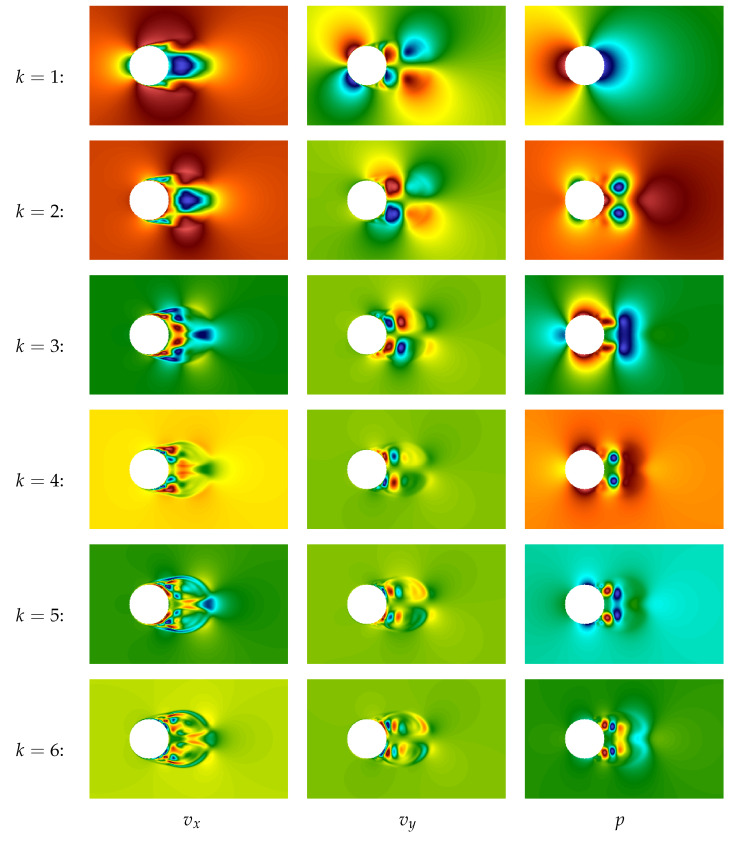
The first six POD-modes for the unsteady flow simulation around impulsively started circular circle at Re=3000.

Plots of the first six POD-modes are shown in Figure 27. On the modes that corresponds to velocity components, large vortex structures are clearly seen, and the larger is number of the mode, the smaller scale of structures it describes.

**Figure 28:**
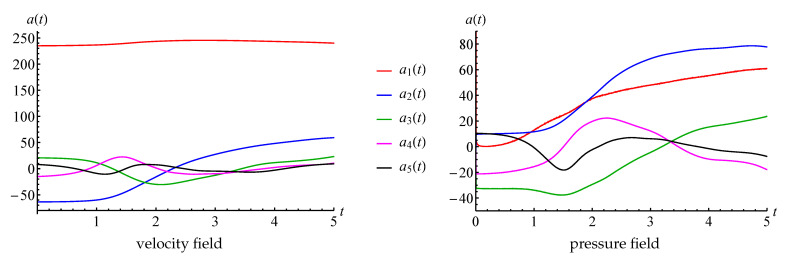
Time coefficient dependencies $a_{k}\left(t\right)$ for first five POD-modes for the flow simulation around impulsively started cylinder at Re=3000.

This flow is essentially unsteady, and it can be considered as a typical transient regime that is especially interesting in engineering practice due to significant variations of the hydrodynamic loads, acting on the structure in the flow. Time dependencies for the coefficients $a_{k}\left(t\right)$ that correspond to the first five POD modes are, consequently, non-periodic (in fact, the same behaviour is observed for coefficients with higher indices), Figure 28.

At the same time, the velocity and pressure fields can be reconstructed with rather high accuracy that seems to be quite enough for practical purposes by taking into account only small number of the POD-modes. The errors for both fields reconstruction are shown in the Table 7 for this simulation; it should be noted that the error decreases not as rapidly as in Table 6 for the quasi-steady flow regime.

As the result, we should point out that even for transient flow regime only 10–20 POD-modes are required for unsteady flow reconstruction with high accuracy: 1% error is achieved for 12 modes. Note that the pressure field is reconstructed with higher accuracy in comparison to velocity field.

In the present simulation, spatial mesh on which velocities were snapped consists of about 60,000 nodes, so in order to store 15 POD-modes, less then 25 Mb of disk space is required; files with time coefficient values $a_{k}\left(t\right)$ are much smaller and their total size is comparable with a size of a file that corresponds to one POD-mode.

### 8.7. Flow Simulation around Two Closely Spaced Circular Airfoils

To verify the accuracy of the unsteady hydrodynamic loads calculation in the VM2D a series of model problems of the external flow simulation around two circular airfoils with different mutual positions were considered. Note that similar problems arising in pipe bundles dynamic simulation, was successfully investigated by using some simplified modification of the vortex method [97].

**Figure 29:**
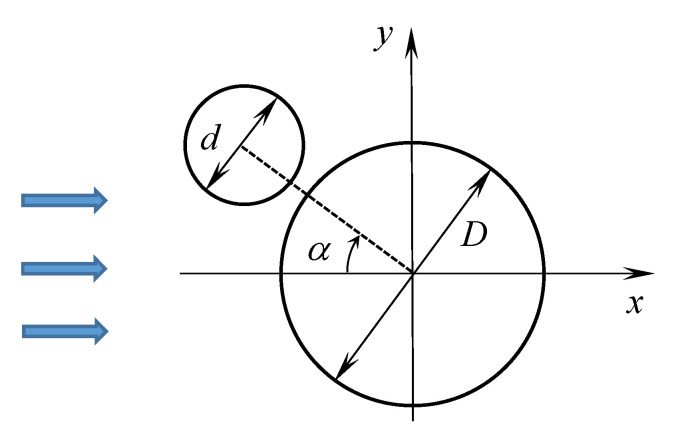
Mutual position of two circular cylinders with different diameters.

Let us consider two closely spaced circular airfoils (Figure 29), the angle α varies from $0^{\circ }$ to $180^{\circ }$, as in [98], where numerical and experimental results for such problems are presented. The incident flow has unit velocity $V_{\infty }=1$, its density ρ=1. The simulations were performed for the regime with intermediate Reynolds number, namely Re=103. The Reynolds number is calculated with respect to the diameter of a large cylinder: Re=DV∞/ν.

In addition to the VM2D, the same simulations were performed in the OpenFOAM. For computations in OpenFOAM meshes with different number of cells were used: 50,000, 150,000, 450,000 cells. Computations in VM2D were performed with different discretization: the large circle was discretized into 250, 500 and 1000 elements (panels), which corresponds to approximately 15,000, 30,000 and 100,000 vortex elements in the vortex wake in steady-state mode, respectively.

The results obtained with different discretization show that for both codes the most coarse discretization is sufficient to obtain results that are in acceptable agreement with the results of two-dimensional simulation in [98]. So in the VM2D code the following discretization has been used: the large circle was discretized into 250 panels, the small one—into 112 panels, the time step was chosen as Δt=0.008.

The following integral characteristics were measured in numerical simulation: the average values and the root mean square (RMS) amplitudes for the drag force coefficient $C_{D}$ and the lift force coefficient $C_{L}$ and the dimensionless vortex shedding frequency St. The drag and lift force coefficients for the large cylinder are calculated as $C_{D1}=\frac{2F_{D1}}{\rho DV_{\infty }^{2}}$ and $C_{L1}=\frac{2F_{L1}}{\rho DV_{\infty }^{2}}$, respectively, and those for the small cylinder $C_{D2}=\frac{2F_{D2}}{\rho dV_{\infty }^{2}}$ and $C_{L2}=\frac{2F_{L2}}{\rho dV_{\infty }^{2}}$, respectively, where $F_{D}$ and $F_{L}$ are the drag and lift forces acting on the cylinder in the horizontal and vertical direction, respectively, the subscripts “1” and “2” denote the large and small cylinders, respectively.

**Figure 30:**
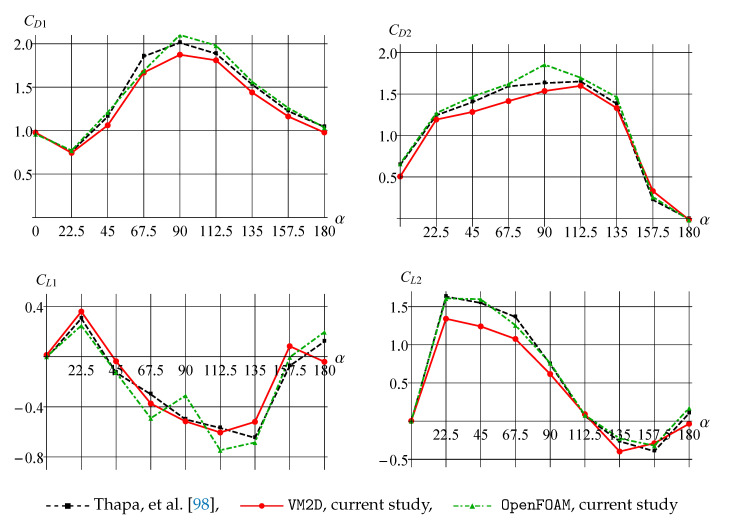
Average values of drag and lift force coefficients $C_{D}$ and $C_{L}$ for large and small cylinders.

In Figure 30, average values of drag and lift force coefficients are shown for different values of α in comparison to the data presented in [98].

It is seen that the results obtained in OpenFOAM and VM2D are in acceptable agreement with the data given in [98]. The largest difference between VM2D and results from [98] is observed for the lift coefficient for the small cylinder $C_{L2}$, while the graph for OpenFOAM is nearly the same as the results [98]. However, for a large cylinder, the results for $C_{L1}$ obtained in VM2D and [98] correlate well enough, while OpenFOAM gives a notable error and incorrect tendency of dependence on the angle.

**Figure 31:**
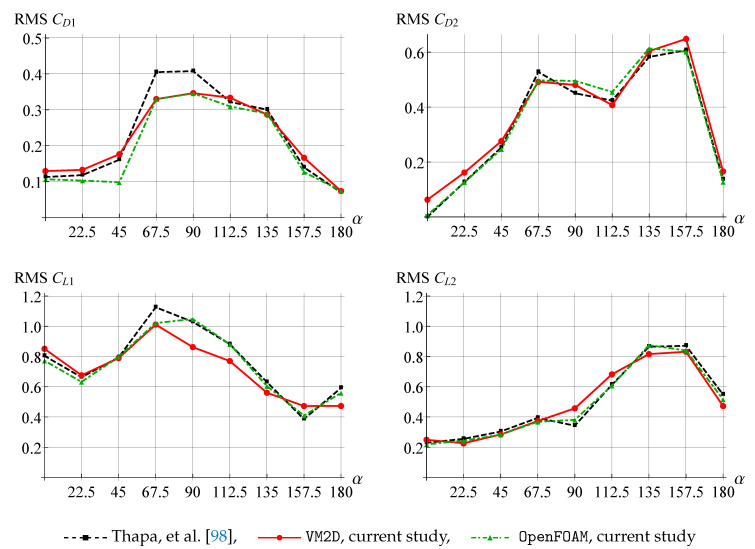
The root mean square (RMS) amplitudes for drag and lift coefficients $C_{D}$ and $C_{L}$ for large and small cylinders.

In Figure 31, the root mean square (RMS) amplitudes for the drag coefficient $C_{D}$ and the lift coefficient $C_{L}$ are shown for different values of α in comparison to the data given in [98].

The RMS amplitude results are also in good agreement for all the experiments. Note only a slight overestimation of the RMS amplitude of $C_{D1}$ obtained in [98] for α=67.5 and α=90.0 in comparison to results from VM2D and OpenFOAM and slight underestimation of the RMS amplitude of $C_{L1}$ for α=90.0 and α=112.5 obtained in VM2D in comparison to results from OpenFOAM and [98].

**Figure 32:**
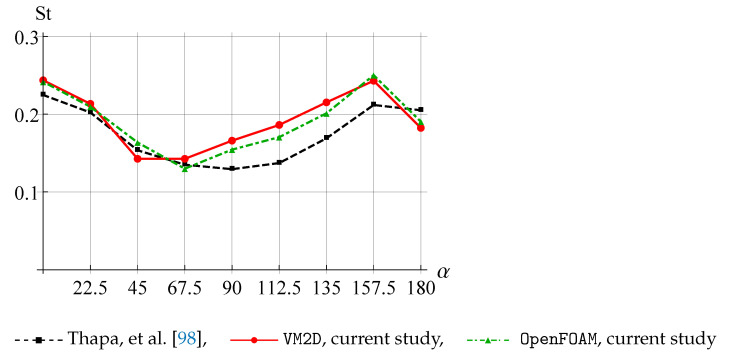
The dimensionless vortex shedding frequency.

The vortex shedding frequency is determined by applying the Fast Fourier Transform (FFT) to the lift coefficients time dependency for the large cylinder and choosing the dominant frequency from the spectra. The dependency of the Strouhal number, calculated as $\mathrm{St}=fD/V_{\infty }$, where *f* is the dominant frequency of the oscillation of the lift coefficients, is shown in Figure 32.

It is seen that the results obtained in VM2D and OpenFOAM agree with each other quite well but slightly differ from the results [98].

As the result, we can conclude that the vortex methods allow not only for average (or steady) hydrodynamic loads computation with acceptable accuracy but also for their time oscillations. This fact is important for engineering applications having in mind the possibility of flow-induced vibrations simulation.

### 8.8. Flow around Rectangular Airfoils

The last model problem is connected to flow simulation around rectangular airfoils with different chord to thickness (c/t) ratio. In the results presented in [95] the interesting phenomenon is observed: the Strouhal number, calculated with respect to rectangular airfoil chord $\mathrm{St}=fc/V_{\infty }$, where *f* is the dominant frequency in the lift force spectra, $V_{\infty }$ is incident flow velocity, depends on the chord length *c* stepwise: for small elongation 3<c/t<5.5 its value is approximately constant, St≈0.5; for 5.5<c/t<9 it varies just slightly around the value St≈1.0, and for rectangles of large elongation 9<c/t<10 it is close to St≈1.5.

**Figure 33:**
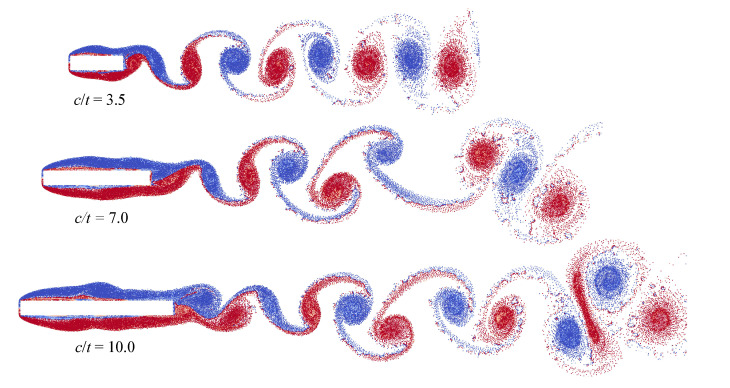
Vortex wakes in quasi-steady regimes for rectangular airfoils with chord to thickness ratios c/t=3.5, c/t=7.0 and c/t=10.0 at the Reynolds number Re=400.

Note that similar behaviour is observed for different Reynolds numbers, we consider the case that corresponds to Re=400 (note that the Reynolds number is calculated with respect to the airfoil thickness, Re=tV∞/ν, where ν if the kinematic viscosity of the flow). The following parameters were chosen in all numerical simulations in VM2D: incident flow velocity $V_{\infty }=5.0$, its kinematical viscosity ν=0.0125, maximal vortex particle strength $\Gamma_{\max}=0.05$, time step Δt=0.002. All the airfoils had thickness t=1 while their chords were in the range 3.5≤c≤10. Vortex wakes in quasi-steady regimes at time moment $t_{*}=100$ are shown in Figure 33.

**Figure 34:**
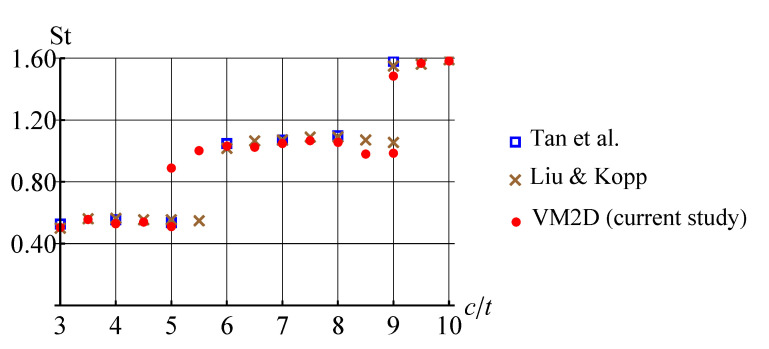
The Strouhal number against chord to thickness c/t ratio at the Reynolds number Re=400.

The obtained values for the Strouhal number are shown in Figure 34 in comparison to results of numerical simulations by Tan, et al. [99] and Liu and Kopp [95] performed also at Re=400.

**Figure 35:**
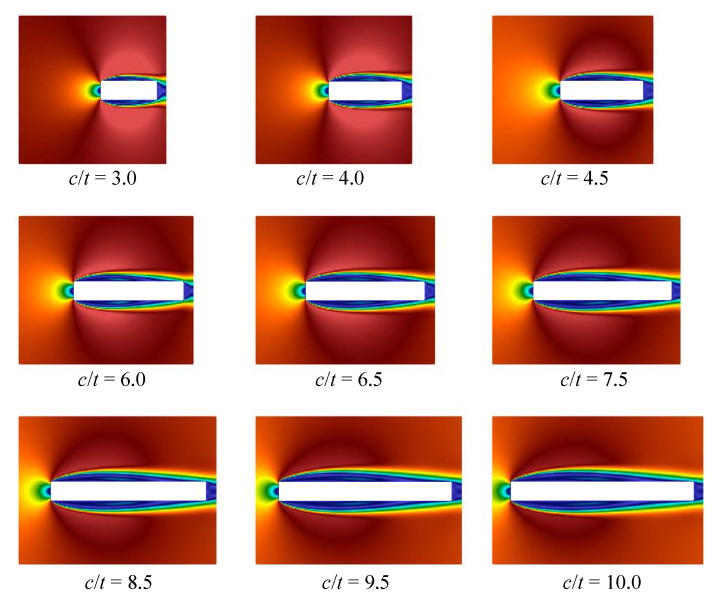
The first POD-mode for velocity field (velocity magnitude is shown) for the quasi-steady flow regime around rectangular airfoils at Re=400.

**Figure 36:**
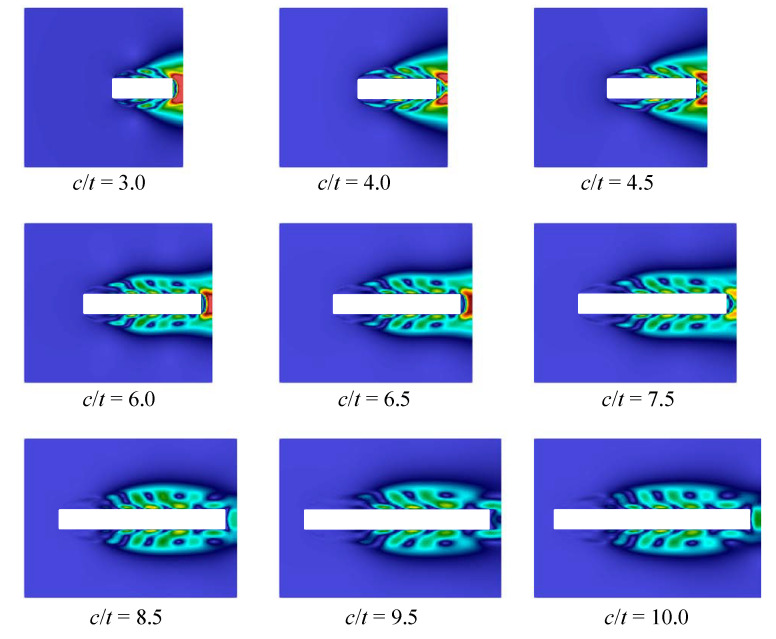
The second POD-mode for velocity field (velocity magnitude is shown) for the quasi-steady flow regime around rectangular airfoils at Re=400.

**Figure 37:**
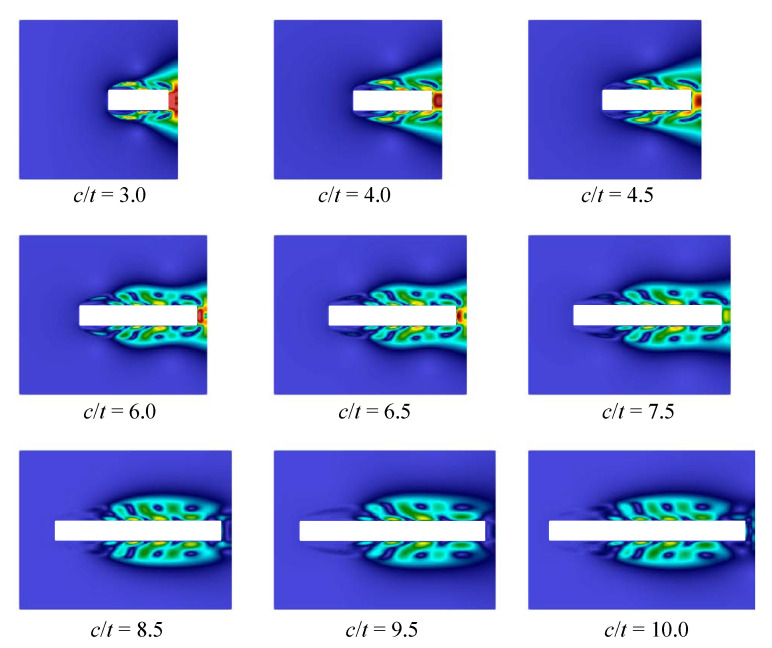
The third POD-mode for velocity field (velocity magnitude is shown) for the quasi-steady flow regime around rectangular airfoils at Re=400.

In [95], it is noted that at c/t=9.0 two dominant frequencies in the spectra are observed, the corresponding Strouhal number values are marked on the plot by crossings. In the present study, a similar situation took place also for c/t=5.0. In general, the results obtained by using the vortex method are in good agreement with the previously known results of numerical simulation. However, it is not easy to recognize the cause of stepwise Strouhal number behavior, watching vortex wake structures. At the same time, the POD-analysis allows for doing it easily. If the POD technique is applied for snapshots processing in quasi-steady regime, i.e., omitting the transient regime after simulation start, during which boundary layer and vortex wake are developing, the following results are obtained for first three POD-modes (Figure 35, Figure 36 and Figure 37).

The first mode corresponds and describes with high accuracy averaged flow, while on the plots for the second and third modes vortex structures are clearly distinguished. These vortex structures have the largest scale; the higher POD-modes correspond to smaller-scale vortices, which accumulate, of course, a much smaller fraction of energy. It is seen that for airfoils with small elongation 3≤c/t≤4.5 two pairs of vortices are formed along their chord, for the airfoils with 6≤c/t≤7.5 three pairs of vortices are observed, and for 8.5≤c/t≤10 four pairs are distinguished. It is interesting also to note that the distance, at which the large-scale vortices are generated in the boundary layer, increases with the c/t ratio.

So, the POD analysis allows for clear distinguishing of different flow regimes that determine macroscopic flow parameters, such as the Strouhal number.

## 9. Conclusions

The possibilities of applying two-dimensional Lagrangian vortex methods of computational fluid dynamics, namely, the particular modification—the Viscous Vortex Domains method—were considered in relation to various types of problems. The original method of Viscous Vortex Domains, developed by prof. G. Ya. Dynnikova, supplemented by the original high-accurate numerical schemes for simulating vorticity generation on the airfoil surface line and implemented in the parallel code VM2D.

All the considered numerical simulations were performed by using the VM2D code. The results of the simulations in the model problems allow us to conclude that the viscous vortex domains method can be efficiently applied to viscous incompressible flows simulation for regimes with low Reynolds numbers when the contribution of three-dimensional effects in the flow is not significant. Simulation for higher Reynolds numbers is also possible, the numerical results are in good agreement with the results of other researchers, however, it should be taken into account that the two-dimensional formulation in this case can be not correct. For example, in the case of bluff airfoils, when considering two-dimensional formulation, the flow separation point is shifted against its true position being observed in the experiment when the Reynolds number is higher than 160. However, for the case when there is an angle point or a sharp edge (i.e., the point from which flow separation occurs in most modes), the range of Reynolds numbers, when the two-dimensional modeling remains correct, can be much wider in comparison to airfoils with a smooth surface line.

For the model problems, the VM2D code verification is performed by computing unsteady hydrodynamic loads acting on the airfoils—the most common application of vortex methods. The simulation results are in good agreement with the numerical results of other authors and experimental data.

The POD technique is used to store and analyze the results of simulations obtained in VM2D which initially have the form of snapshots (in VTK format) at different time steps, containing information about the velocity and pressure on the spatial mesh. The POD application makes it possible to reduce significantly the amount of memory required for data storing. Of course, such data compression comes with some loss and it is impossible to reconstruct the original physical fields exactly, but the error analysis shows that the storing of 10–20 first POD-modes (instead of hundreds or thousands of original snapshots) is quite enough to provide about one percent error.

From the above, the authors are pleased to conclude that they managed to create a computational tool that allows for flow modeling using pure Lagrangian vortex methods, which is available to researchers, first of all, to engineers. The performed extensive verification confirms its efficiency and applicability in a wide range of problems.

## Figures and Tables

**Table 1:** Relative errors of the added masses tensor estimation for the wing airfoil for different numerical schemes.

Number of Panels	*N*-Scheme (DVM)	$T^{0}$-Scheme (Const)	$T^{1}$-Scheme (Linear)
50	$3.4\times 10^{-1}$	$5.7\times 10^{-2}$	$1.7\times 10^{-2}$
100	$6.2\times 10^{-2}$	$2.5\times 10^{-2}$	$4.6\times 10^{-3}$
200	$3.9\times 10^{-2}$	$1.3\times 10^{-2}$	$1.7\times 10^{-3}$
400	$2.2\times 10^{-2}$	$6.9\times 10^{-3}$	$7.4\times 10^{-4}$
800	$1.2\times 10^{-2}$	$3.7\times 10^{-3}$	$3.5\times 10^{-4}$
1600	$6.1\times 10^{-3}$	$2.0\times 10^{-3}$	$1.7\times 10^{-4}$
3200	$3.2\times 10^{-3}$	$1.1\times 10^{-3}$	$8.5\times 10^{-5}$

**Table 2:** Relative errors of the added masses $\lambda_{xx}$, $\lambda_{xy}$, $\lambda_{x\omega }$ estimation for the wing airfoil for the $T^{1}$-scheme.

N	50	100	200	400	800	1600	3200
$\delta_{x}$	$1.7\times 10^{-2}$	$4.6\times 10^{-3}$	$1.2\times 10^{-3}$	$2.97\times 10^{-4}$	$7.5\times 10^{-5}$	$1.9\times 10^{-5}$	$4.7\times 10^{-6}$

**Table 3:** Number of panels proving the same accuracy of added masses estimation for the wing airfoil for different numerical schemes.

	*N*-Scheme (DVM)	$T^{0}$-Scheme (Const)	$T^{1}$-Scheme (Linear)
1%	950	265	66
0.1%	10,800	3700	307

**Table 4:** Relative errors of the added masses tensor estimation for elliptical airfoil with a=1.0 and b=0.2 semiaxes for different numerical schemes.

Number of Panels	*N*-Scheme (DVM)	$T^{0}$-Scheme (Const)	$T^{1}$-Scheme (Linear)
50	$1.3\times 10^{-1}$	$6.1\times 10^{-2}$	$3.2\times 10^{-2}$
100	$4.4\times 10^{-2}$	$1.8\times 10^{-2}$	$1.0\times 10^{-2}$
200	$1.3\times 10^{-2}$	$4.7\times 10^{-3}$	$2.8\times 10^{-3}$
400	$3.4\times 10^{-3}$	$1.2\times 10^{-3}$	$7.3\times 10^{-4}$
800	$8.7\times 10^{-4}$	$2.9\times 10^{-4}$	$1.8\times 10^{-4}$
1600	$2.2\times 10^{-4}$	$7.3\times 10^{-5}$	$4.6\times 10^{-5}$
3200	$5.6\times 10^{-5}$	$1.8\times 10^{-8}$	$1.2\times 10^{-5}$

**Table 5:** Separation angle values against Reynolds numbers.

Re	20	30	40	50
$\bar{\theta }$	140.9	133.9	125.7	122.0
$\theta_{min}\ldots \theta_{max}$	—	—	—	—
**Re**	**60**	**80**	**100**	**120**
$\bar{\theta }$	119.5	119.2	117.2	117.2
$\theta_{min}\ldots \theta_{max}$	118.3…122.6	116.7…125.8	115.7…124.4	114.66…123.66
**Re**	**140**	**160**	**180**	**200**
$\bar{\theta }$	117.2	119.4	120.7	119.7
$\theta_{min}\ldots \theta_{max}$	113.9…121.5	113.2…121.1	112.5…122.2	111.4…124.0

**Table 6:** The error of velocity and pressure fields reconstruction for different number of the POD-modes.

m	1	3	5	7	9	11	13	15	17	19
$\delta_{V}^{\left(m\right)}$	0.3370	0.0749	0.0417	0.0180	0.0098	0.0066	0.0049	0.0039	0.0036	0.0034
$\delta_{p}^{\left(m\right)}$	0.3111	0.1118	0.0240	0.0123	0.0052	0.0029	0.0022	0.0016	0.0013	0.0011

**Table 7:** The error of velocity and pressure fields reconstruction for different number of the POD-modes.

m	2	4	6	8	10	12	14	16	18	20
$\delta_{V}^{\left(m\right)}$	0.0913	0.0390	0.0255	0.0182	0.0130	0.0101	0.0080	0.0064	0.0053	0.0046
$\delta_{p}^{\left(m\right)}$	0.0139	0.0044	0.0019	0.0011	0.0006	0.0004	0.0003	0.0002	0.0002	0.0001
